# Isotopic data reveal a localist Roman population in late Roman *Albintimilium*, Liguria

**DOI:** 10.1038/s41598-025-92851-7

**Published:** 2025-04-09

**Authors:** Sarah Defant, Alessandro Carabia, Rafał Fetner, Elizabeth Craig-Atkins, Ricardo Fernandes, Gian Piero Martino, Stefano Costa, Arkadiusz Sołtysiak, Adam Izdebski

**Affiliations:** 1 Institute of Prehistoric Archaeology, Freie Universität Berlin, Fabeckstr. 23/25, 14195 Berlin, Germany; 2 Max Planck Institute of Geoanthropology, Kahlaische Straße 10, 07743 Jena, Germany; 3 School of History, Classics and Archaeology, Newcastle University, Armstrong Building, Newcastle upon Tyne, NE1 7RU UK; 4 Department of Bioarchaeology, Faculty of Archaeology, University of Warsaw, ul. Krakowskie Przedmieście 26/28, 00-927 Warsaw, Poland; 5 School of History, Philosophy and Digital Humanities, The University of Sheffield, Western Bank, Sheffield, S10 2TN UK; 6 Former Soprintendenza per i Beni Archeologici della Liguria, La Spezia, Italy; 7 Soprintendenza Archeologia, Belle Arti e Paesaggio per le Province di Imperia e Savona, Via Balbi 10, 16126 Genoa, Italy; 8 Faculty of Liberal Arts, University of Warsaw, ul. Nowy Świat 69, 00-046 Warsaw, Poland

**Keywords:** Archaeology, Biological anthropology, Stable isotope analysis, Biogeochemistry, Environmental sciences

## Abstract

This study investigates human diet and mobility to understand the socio-economic organisation of a Late Roman community in Liguria, a transitional region between Italy and Gaul, during the 3rd–5th century CE. By combining archaeological, historical, osteological, and isotopic data with novel Bayesian modelling of multi-isotope data (collagen δ^13^C, δ^15^N, bioapatite ^87^Sr/^86^Sr) from human and animal skeletal remains, as well as modern plant samples, we provide new insights into this hitherto under-researched region. Our findings suggest the community followed a C_3_-based diet, heavily reliant on plant resources and carbohydrates, supplemented by animal protein, likely from omnivorous pigs. This characteristically Roman diet contrasts with ancient written sources that claimed Ligurians had a “barbarian” diet and lifestyle. We also identified significant sex-based dietary differences, with men consuming more animal-derived protein than women, reflecting traditional Graeco-Roman societal ideals. Although the overall dietary pattern aligns with Roman norms, there is no isotopic evidence of long-distance migration or consumption of significant amounts of imported food. This indicates that the community may have been more localist, prioritising locally available resources over long-distance imports, which is unexpected given the prevalent idea of a large-scale interconnected food network within the Roman Empire.

**Supplementary Information:**

The online version contains supplementary material available at 10.1038/s41598-025-92851-7.

## Introduction

Between the 3rd and early 5th century CE, the Western Roman Empire underwent multiple phases of crisis and recovery marked by the interventions of a series of energic rulers such as Diocletian and Constantine I. In this period, the coastal area today known as Liguria and the town of *Albintimilium* (modern Ventimiglia) followed the general political and economic trends of crisis and recovery of Northern Italy but remained mostly on the edges of the political and military struggles. Liguria and the *municipium* of *Albingaunum* (modern Albenga) appear in the Historia Augusta (*Hist. Aug. XVIII*) as the birthplace of Proculus, one of the military emperors of the 3rd century. When *Mediolanum* (modern Milan) was designated as the new imperial capital by Diocletian in 284 CE, Liguria—especially Genoa—gained significance as an important maritime access point for the new capital^1^. Despite the growing insecurities of the northern Italian regions towards the end of the 4th century, Liguria seems to have remained mostly untouched, possibly due to its more marginal position on the northern Italian frontier. It was only during the early 5th century that it came under direct threat by the Visigothic incursions, who sacked Rome in 410 and retreated northwards, possibly crossing the region in their march towards southern France^2^. It is in this context that the general Constantius (future emperor Constantius III) restored and re-fortified the *municipium* of *Albingaunum* in 410–420 CE^3^. The economic prosperity of the region is usually connected to the fortunes of the ancient Tyrrhenian maritime route, which connected North Africa and Southern Italy with Southern France and Spain^4^. This route was continuously active in Antiquity, with varying levels of traffic, and was revitalised by the aforementioned designation of Milan as one of the capitals of the Empire, a title kept until 402 CE^5^.

**Fig. 1:**
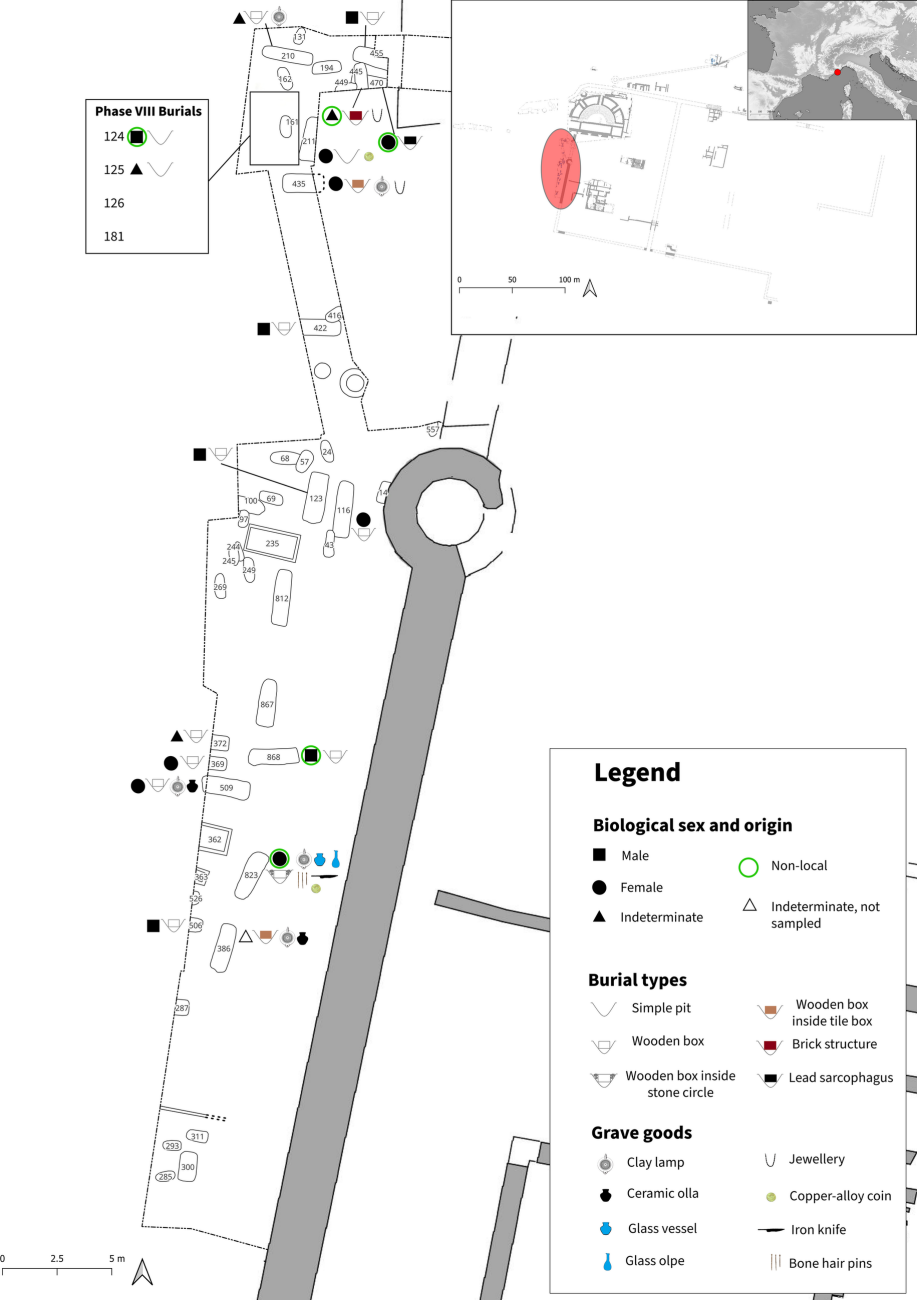
Plan of the “Necropoli del Sottopasso” (location of the site shown in the inset top right corner) showing the burials of both phases and the main characteristics of the analysed depositions. For Phase VIII, only the general area of discovery is indicated on the map since the exact location of the graves is unknown.

*Albintimilium*, which expanded from the older Ligurian *oppidum* of *Albium Intemelium*, remained one of the main settlements of the region despite being seldomly mentioned in the sources of this time. The city, which started as a military *castrum* and gradually developed into a Roman city with paved roads, public buildings, and stone walls, is mentioned in the 3rd -early 4th century CE *Itinerarium Maritimum*, with its port defined as *plagia* (beach) (*Itin. Anto. 503.3*), however, most of the information comes from archaeological excavations^6,7^. In the period between the 3rd and the early 5th century the urban framework is marked by a general continuity: the road networks and the buildings of the Imperial phase were generally well maintained and continued to serve their original function^7^. Marked signs of a change are visible only after the first decades of the 5th century, when all known public buildings, such as the theatre^8^, aqueduct, and baths, were abandoned^9^. Private houses were in ruins or had their rooms subdivided into smaller and simpler spaces^6^. From the late 5th or early 6th centuries, scattered inhumations are attested in the settlement between the theatre, the *decumanus*, and the baths (Fig. 1), further testifying to the new urban framework typical of Late Antiquity^10^. The Justinianic reconquest of Italy and the presence of imperial troops in the region may have initiated a population shift to the more defensible Cavo Hill—the future nucleus of the medieval town (1.5 km to the West), already in the 6th century^11^. The city then once again became a border area, this time between the Eastern Romans, the Longobards, and the Franks, suffering the effects of the Longobard military conquest of the mid 7th century (Fred., *Chron.*, 71; Pal. Deac., *Hist. Lang.*, IV.45). *Albintimilium* was likely abandoned during the 7th or 8th centuries in favour of the hilltop’s more defensible position.

There is a pronounced scarcity of literary sources focusing on this region and time period, but archaeological research has revealed typical Late Antique changes in the urban landscape. Yet our understanding of the population residing in the settlement and its surrounding region during the transitional period between the 3rd and 5th centuries CE remains limited. The constraints of traditional historical and archaeological approaches underscore the necessity of our research, which focuses on defining dietary and mobility patterns, as a means of enhancing our understanding of lived experiences during this age of societal change and large migration movement.

While there have been extensive studies of past dietary and mobility patterns across Europe through stable isotope analysis, significant gaps remain for Late Antiquity. In Italy, much attention has been given to the Longobards^12–14^, but non-Longobard areas and specific periods like the Late Roman Empire have been largely overlooked. In Southern France and Northwestern Italy, only a few research projects have examined dietary patterns between the 3rd and 5th centuries CE, with the most relevant data emerging from Southern France^15,16^. The majority of investigations in the western part of the northern Italian Peninsula focus on the transition from Late Antiquity to the Early Middle Ages or the Longobard period, often through diachronic studies comparing Late Roman and Early Medieval populations (e.g., in Central Italy/Tuscany^17^ and various regions of Northern Italy^14^). Only two studies have included strontium isotope analysis to investigate mobility^14,18^, and none have focused on Late Roman Liguria. Therefore, our research represents the first comprehensive study of dietary and mobility patterns of the inhabitants of this hitherto under-researched part of the Italian Peninsula. We achieve this by integrating dental pathology of dietary aetiology and multi-isotope data (carbon, nitrogen and strontium) with osteological, archaeological, historical, and paleoenvironmental data. Through this approach, we aim to illuminate potential social dynamics related to resource distribution and migration, and assess the impact of imported foodstuffs, possibly facilitated by *Albintimilium’*s maritime connections with the Mediterranean^19–22^, on the community’s diet compared to locally available products.

### Dental pathologies as dietary indicators

We collected data on dental pathologies, which have been used extensively as proxies for past dietary patterns^23–26^ and, therefore, provide valuable comparative information to the isotopic analyses. Caries, the progressive demineralisation of the enamel, dentine and cement, is caused by organic acids which are produced during the fermentation of carbohydrates. It is, therefore, often associated with a high consumption of fermentable carbohydrates, while proteins and fats do not tend to play a significant role in the formation of carious lesions. In contrast, calculus, as the mineralised or calcified form of dental plaque, is often associated with protein-rich diets, although it does not necessarily indicate a certain diet, as a variety of factors such as individual variation, oral hygiene habits, age, systemic disease, mineral content in drinking water or cultural practices influence the formation of calculus^27–30^. Nonetheless, it can be used as supplementary information since a diet that is high in protein leads to a more alkaline oral environment and, therefore, leads to a faster accumulation of dental plaque on the teeth^23,28^.

### Isotopic approaches to diet and mobility

The most common method used in archaeological research to reconstruct dietary patterns in past communities is through stable isotope analysis of carbon and nitrogen of collagen extracted from bone or teeth. It reflects a mixture of isotopic signatures of consumed foods and can, therefore, aid in interpreting past dietary patterns^31–35^. The measured isotopic ratios of carbon and nitrogen in bone collagen predominantly reflect the protein fraction of the diet^36,37^. Carbon stable isotopes (δ^13^C) measured in collagen are used to assess the protein contributions from plants following different photosynthetic pathways (C_3_, C_4_ or CAM plants) and of the animals that consumed these^31,35,38,39^. Nitrogen stable isotopes (δ^15^N) reflect the trophic level of the dietary protein consumed, increasing by approximately 3–6‰ at each step up the food chain^35,37,40–44^. Environmental factors such as aridity, agricultural practices, and soil salinity can influence both δ^13^C and δ^15^N plant values^45^.

For accurate interpretations of human isotopic values and correct positioning within their ecosystems, having a representative food isotopic baseline is imperative^32,37^.

We utilised strontium isotopes ratios (^87^Sr/^86^Sr) to investigate spatial mobility in the population of *Albintimilium* through the comparison of isotope values from the inorganic component of teeth to the local environment. Strontium isotope ratios vary with age and composition of bedrock and are further influenced by environmental factors (e.g. sea spray, sand-blown dust, precipitation)^46–48^. The ^87^Sr/^86^Sr ratio in human enamel reflects the dietary strontium consumed during tooth formation. By comparing the values obtained from human enamel samples to the established bioavailable strontium baseline for the area around where the remains were recovered, it is possible to assess whether an individual falls within the local range of values or should be considered an immigrant^49,50^. It is essential to acknowledge that variability in geological and environmental features within the defined local area, overlap between local signatures and those from geographically distant areas^49–51^, and the consumption of large quantities of imported resources from regions with different isotopic signatures^46,52^ may also introduce complexities obscuring definitive identification.

## Material: the “Necropoli Del Sottopasso”

Roman necropoleis were typically located outside the city gates along the main roads. In *Albintimilium*, the only Roman necropolis so far discovered was located on the western axis of the via Iulia Augusta, leaving the city towards southern Gaul^53^ (Supplementary Note S1). The continuity of use of this area between the cremations of the Late Republican and Early Imperial phases and the inhumation practices of the Late Empire is marked by the discovery of the “Necropoli del Sottopasso” (Supplementary Note S1 and Supplementary Fig. S3)^54,55^. Before this, only a few inhumations were known in the same area^8,10,53^. The “Necropoli del Sottopasso” (Fig. 1 and Supplementary Fig. S1), located just outside the western city walls, was the first consistent nucleus of Late Roman/Late Antique burials in *Albintimilium* excavated following a modern stratigraphic methodology. It was investigated between 1989 and 1993 under the direction of Gian Piero Martino and was preliminarily dated between the 3rd and 5th centuries CE. The site remained the only consistent inhumation necropolis for the *municipium* until the more recent discovery of a new graveyard placed just outside the northern city walls, dated to the 6th and 7th centuries. The new site is still under investigation, and it was not possible to include it in the present research^56^.

The area of the “Necropoli del Sottopasso” covered a narrow strip approximately seven or eight metres wide and forty metres long, running north-south parallel to the western city walls. The excavators divided it into four main sectors, with seventeen “trenches” (*interventi*) (Supplementary Fig. S2). Overall, archaeologists identified nine main phases of occupation in the area, with the necropolis linked to Phases VII and VIII. Phase VII, after the deposition of new soil to level the area, saw the establishment of the graveyard, which remained in use between the 3rd and 4th centuries. Phase VIII is marked by the natural deposition of a first layer of sand across the area, suggesting a hiatus in the use of the space. This was followed by the deposition of only two individuals (124, 125), plus the fragments of possibly two more (126, 181), all in trench six, which then marked the end of funerary use of the area. The absence of grave goods in Phase VIII has prevented any precise dating of this later usage. The new radiocarbon dates of individual 125 now indicate that Phase VIII most probably falls some time between the end of the 4th and the beginning of the 5th century CE (for the detailed ^14^C results, see Supplementary Note S5 and Supplementary Figs. S22–S24). The area was then abandoned and saw the spoliation of the city walls and the natural deposition of a thick layer of aeolian sand, the same that covered most of the Roman city after its abandonment^6^.

The excavation revealed 50 burials with remains of at least 46 individuals (Fig. 1). While most graves were simple pits with or without wooden boxes, there was also a large number of *enchytrismòs* burials noted, which were however reserved for infants, and not included in the present study. However, there were also other burial types attested, such as monumental structures, “alla cappuccina” (rooftiled tomb), tile boxes, and a lead sarcophagus^57^.

Only nine graves (Supplementary Table S1) presented burial goods, testifying to some weak form of continuity with the older cremation rites, for which the deposition of goods was an essential element (Supplementary Note S1). From the 46 individuals originally reported, 43 skeletons were available for osteological analysis to provide insights into the community’s demographic structure and offer contextual information for the isotopic analyses that facilitate deeper insights into patterns of diet, migration, and lifestyle.

We limited the stable isotope analysis and the analysis of dental pathologies to adolescent and adult individuals who had preserved ribs and teeth (*n* = 18). We included individuals with at least one tooth in the analysis of dental pathologies (carious lesions and calculus depositions). This resulted in a total of 403 permanent teeth for Phase VII individuals and 34 permanent teeth, 1 retained deciduous tooth for Phase VIII individuals being analysed. These 18 individuals were sampled for carbon, nitrogen, and strontium isotope analysis (16 from Phase VII; two from Phase VIII). To support the archaeological dating, two individuals (455 and 125), representing the two phases, were sampled for radiocarbon ^14^C) dating.

The 39 individuals (16 sampled for stable isotopes) from Phase VII should be representative of a period of prosperity, or at least of stability, of the settlement. Decades of excavation showed the renovation of several buildings and uninterrupted relation with the Mediterranean trade system, underlined by the influx of imported materials from North Africa and the eastern Mediterranean^19–21^. The four remaining individuals of Phase VIII (two sampled for stable isotopes) might instead give us a glimpse into the troubled years of the early 5th century.

## Results

### Osteology and archaeological context

**Fig. 2:**
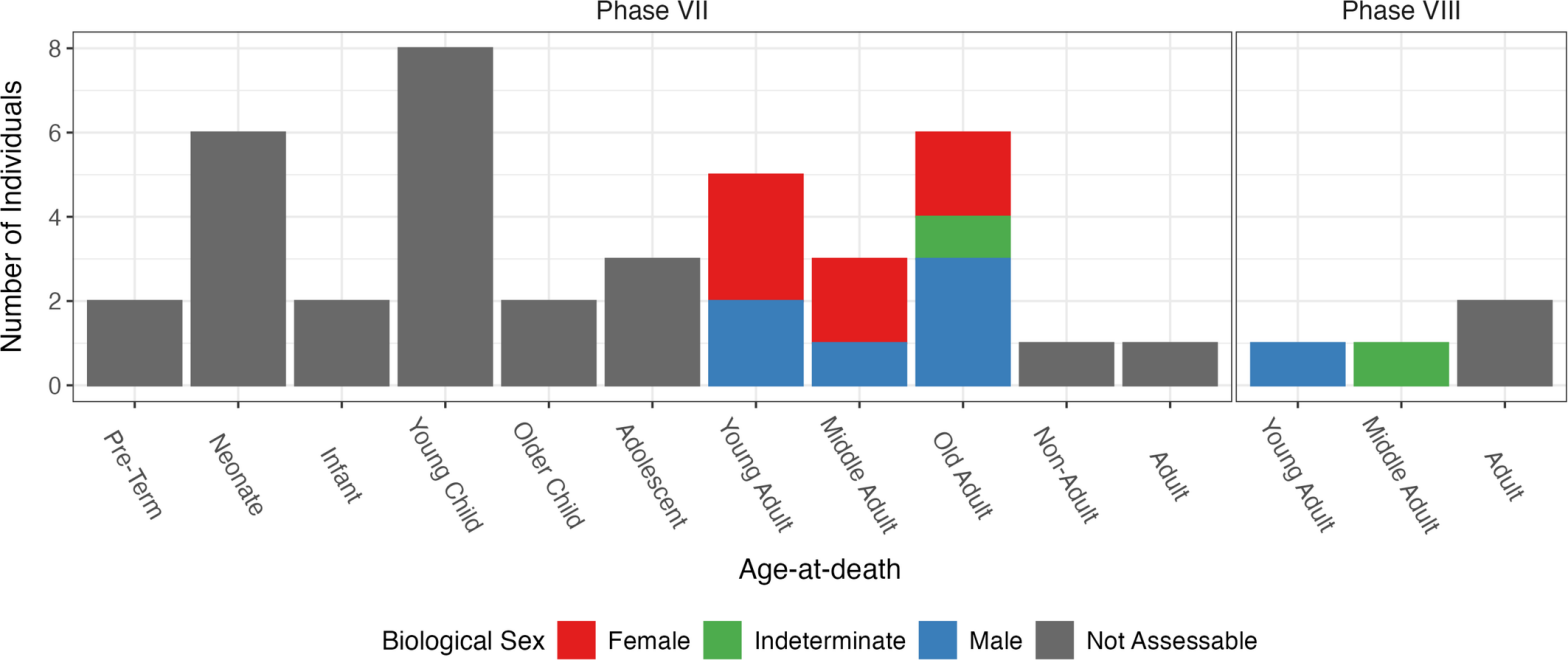
Age-at-death and biological sex distribution of individuals from the “Necropoli del Sottopasso”.

Out of the 43 recovered individuals, 39 were attributed to Phase VII, with the majority of them being categorised as non-adults under the age of twelve (*n* = 23). There were similar proportions of adult males and females in each of the three adult age categories (Fig. 2). The remaining four individuals were attributed to Phase VIII and were all found to be adults. Detailed osteological and archaeological information on each individual can be found in Supplementary Dataset S1.

Among the adolescents and adults, which are the focus of this study, grave goods were attested in seven graves (Supplementary Figs. S4–S14); five of them belonged to females (386, 435, 509, 823, 470), while the remaining two (210, 445) were of indeterminate sex. Graves of adult females were also more variable (simple pits, wooden boxes, wooden boxes in tiled boxes and a lead sarcophagus) compared to those of adult males, who were all buried in wooden coffins. There was no clear relationship between age-at-death and funerary depositions.

### Dental pathologies

All statistical analyses pertain only to Phase VII individuals, as there were only two individuals with observable dentitions from Phase VIII. No significant differences were found in distributions of tooth types between sexes (χ^2^ = 0.49, df = 7, *p* = 1). Therefore, all tooth categories were pooled together for further analysis pertaining to prevalence rate of dental pathologies.

### Caries

The overall prevalence of caries was high—13 per 16 individuals (with at least one observable tooth) from Phase VII, and both individuals from Phase VIII had at least one carious lesion. A significant difference was observed between sexes (Phase VII), with females’ teeth (49 (affected) /185 (observable), 26.5%) expressing more carious lesions than males’ teeth (19/143, 13.3%; χ^2^ = 7.77, df = 1, *p* = 0.005).

### Calculus

Dental calculus was found in all 16 individuals from Phase VII and in one of two from Phase VIII. Males’ teeth were more commonly affected by dental calculus (84/140, 60%) than females’ teeth (66/170, 38.8%; χ^2^ = 12.95, df = 1, *p* < 0.001).

### Carbon and nitrogen stable isotopes

A total of 16 out of 18 human samples (Supplementary Dataset S1) and 15 out of 19 animal samples (Supplementary Dataset S2) met the established quality criteria for collagen preservation^58–60^ and were used for further analyses.

### Faunal isotope results

**Fig. 3:**
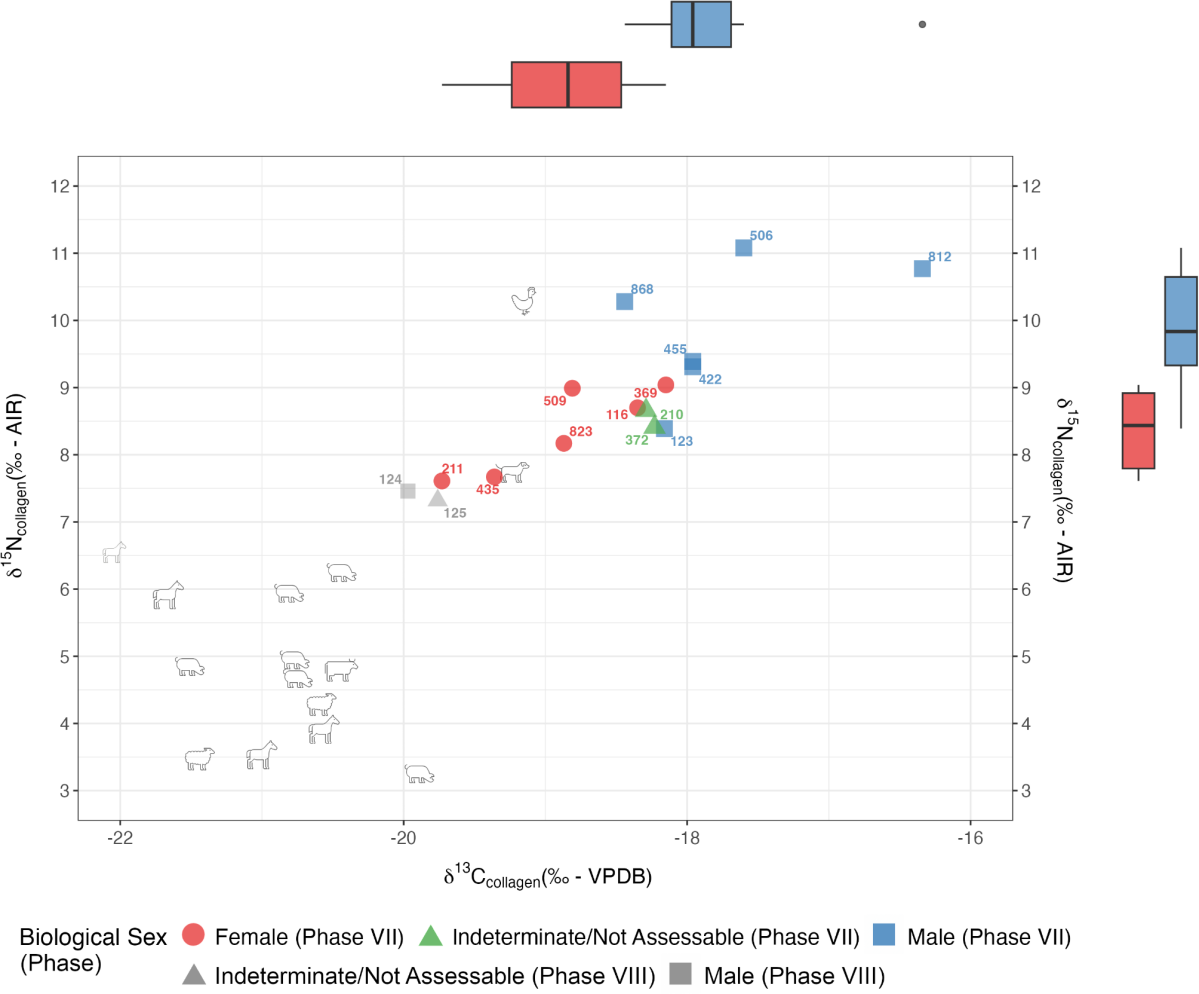
Scatterplot with marginal boxplots showing δ^13^C_collagen_ and δ^15^N_collagen_ values of humans and animals from Albintimilium. Humans are divided into their respective phases and biological sex. The marginal box and whiskers plots represent 68% and 95% ranges of δ^13^C_collagen_ and δ^15^N_collagen_ values in female (red) and male individuals (blue) from Phase VII, with the solid line representing the median. Animals are presented by icons. A juvenile horse sample, excluded from further analysis, is shown with a smaller, lighter icon.

Faunal bulk bone collagen δ^13^C_collagen_ and δ^15^N_collagen_ measurements range from − 22‰ to − 19.2‰ and 3.5‰ to 10.5‰, respectively (Fig. 3). Sampling included taxa commonly eaten as part of the Roman diet (ovicaprid and pig), but also taxa which were attested to have mainly been used as work animals (horses, cattle, and dogs). Overall, the animal values indicate a C_3_ terrestrial diet with only small variation and no evidence of direct C_4_ protein consumption.

The difference in both δ^13^C_collagen_ and δ^15^N_collagen_ values among domesticated ovicaprids, cattle, pigs, dog, and chicken may reflect variations in their diets, specifically in the ratios of plant and animal protein consumed. These differences may also be influenced by the intake of varying proportions of plants following different photosynthetic pathways or by variations in animal husbandry and agricultural practices. Values of pigs, traditionally omnivorous animals, are close to values of herbivores (ovicaprids, cattle, horses), suggesting that their diet contains a large proportion of plant-based foodstuffs. A more detailed discussion of faunal values and their interpretations can be found in Supplementary Note S3.

### Human stable isotope results

Stable carbon and nitrogen isotopic ratios in human bone collagen for Phase VII range from − 20‰ to − 16.3‰ and 7.3‰ to 11.1‰ respectively (Fig. 3). In comparison to animals, humans display an enrichment factor in both carbon and nitrogen values relative to pigs.

Two individuals (506, 812) may be considered outliers based on both the 1.5 Interquartile Range (IQR) of the two variables separately (812 for δ^13^C_collagen_ and 506 for δ^15^N_collagen_) and the bivariate quantiles of the joint distribution when compared to the rest of Phase VII individuals. However, these two individuals—both male—are not flagged as outliers when compared to the remaining male individuals in the sample.

Significant differences were found for both δ^13^C_collagen_ values (Mann–Whitney U test, *p* = 0.020) and δ^15^N_collagen_ values (Mann–Whitney U test, *p* = 0.015) between biological sexes of the main burial phase. Female individuals show both significantly lower median δ^13^C_collagen_ (− 18.8‰) and median δ^15^N_collagen_ (8.4‰) values than males (median δ^13^C_collagen_: − 18.0‰; median δ^15^N_collagen_: 9.8‰). Additionally, male individuals show a broader range of both carbon (2.1‰) and nitrogen (2.7‰) values compared to female individuals (1.6‰ and 1.4‰ respectively) (Fig. 3).

The correlation between δ^13^C_collagen_ and δ^15^N_collagen_ amongst the individuals of Phase VII is strongly positive and significant (*r*_*S*_=0.77, *p* < 0.01), particularly in female individuals (*r*_*S*_=0.94, *p* = 0.02), while in male individuals it is non-significant (*r*_*S*_=0.58, *p* = 0.2).

The values of the individuals attributed to the latest phase (124 and 125) were found at the bottom range of the individuals for both δ^13^C_collagen_ and δ^15^N_collagen_ (Fig. 3).

Although no statistical tests targeting potential socioeconomic or chronological differences could be carried out due to small sample sizes, we were able to make some qualitative observations (Supplementary Fig. S15). Individuals in simple pits had the lowest δ^15^N_collagen_ values amongst the entire community. Individuals buried in wooden coffins showed a relatively wide variety of values, particularly male individuals, who were all buried in wooden coffins. The female individuals buried in potentially more elaborate graves (coffin inside a tile box (435) or coffin surrounded by stones (823)) had slightly lower δ^13^C_collagen_ and δ^15^N_collagen_ values than most other female individuals. From the seven individuals buried with grave goods, only four were available for carbon and nitrogen isotope analysis, and their δ^13^C_collagen_ and δ^15^N_collagen_ values did not appear to follow a different pattern compared to individuals without grave goods. Overall, despite the fact that all males share the same burial type, there is greater variability in diet, while the variability in women is much lower despite a larger variety of funerary rites.

### ReSources model

For dietary caloric estimations, the Bayesian mixing model ReSources^61^ (an updated version of FRUITS^36^) was employed. Model details and model configurations can be found in Supplementary Note S4.

Two primary models were tested. The first one uses five major food groups: C_3_ plants, C_3_ animals, C_4_ plants, freshwater resources, and marine resources. For the second model, the C_3_ animals were further split up into species to narrow down individual contributions, following the example of Cocozza and colleagues^62^, resulting in a total of eight food groups: C_3_ plants, ovicaprids, cattle, pig, poultry, C_4_ plants, freshwater resources, marine resources (Supplementary Fig. S16a,b).

In order to get a representation as accurate as possible for *Albintimilium*, we opted to use only data deriving directly from the site wherever possible. This inevitably reduced the sample size for some taxa significantly (e.g., cattle and poultry). Additionally, a series of priors derived from historical, zooarchaeological, archaeobotanical and ethnographic sources were utilised.

**Fig. 4:**
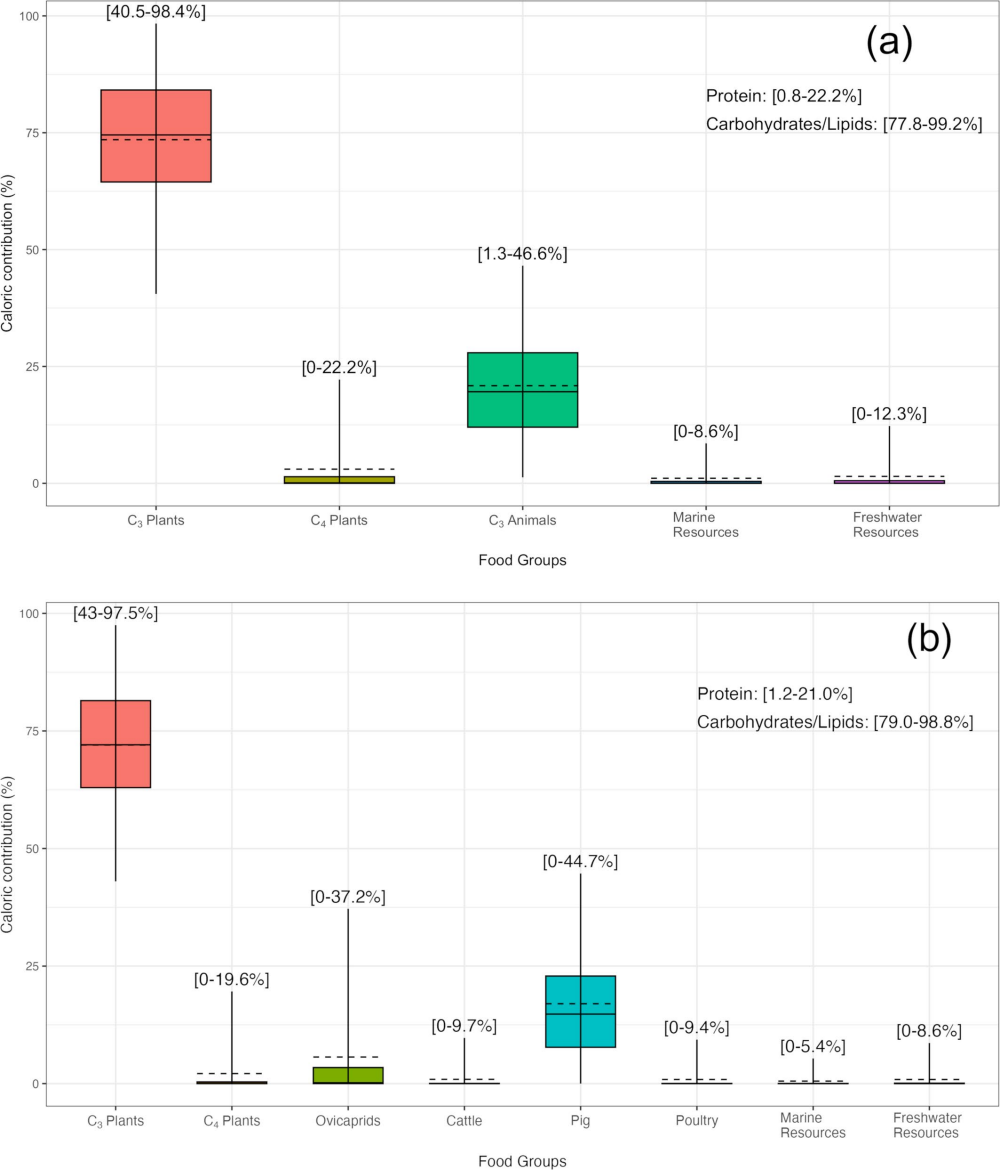
(**a**,**b**) Bayesian estimates of dietary caloric contribution in all human individuals. (**a**) 5 food groups (C_3_ plants, C_4_ plants, C_3_ animals. marine resources, freshwater resources), (**b**) 8 food groups (C_3_ plants, C_4_ plants, ovicaprids, pig, cattle, poultry, marine resources, freshwater resources). Boxes and whiskers represent 68% and 95% confidence intervals, respectively. Horizontal continuous lines represent the mean and dashed horizontal lines the median. Numbers in brackets represent 95% confidence intervals. Also included within the graph are numeric estimates of the caloric contributions of protein versus carbohydrates/lipids macronutrients.

The resulting Bayesian dietary caloric estimates, which represent a group average for the entire sampled community, are shown in Fig. 4a, b and Supplementary Fig. S17a–f. Variations between individuals in the amount of specific resources consumed (e.g. higher proportions of aquatic resources for individuals with higher δ^13^C_collagen_ and δ^15^N_collagen_) are expected and discussed later.

Both models (Fig. 4a,b), representing the average of the entire sampled community, show a high reliance on foodstuffs deriving from C_3_ terrestrial sources, with much lesser contributions from aquatic (marine and freshwater) and C_4_ sources. Additionally, the model revealed that the main source of calories for late Roman *Albintimilium* were C_3_ plants, such as wheat, barley, legumes, fruits, and vegetables, followed by C_3_ animals. The main source of protein, however, most likely were animals consuming C_3_ plants, such as pigs and ovicaprids, followed by C_3_ plants, while C_4_ plants (e.g., millets) and aquatic resources played only marginal roles, if any.

Through splitting up the C_3_ animals into additional categories (Fig. 4b), we were able to observe that individuals consumed larger amounts of protein derived from pigs than ovicaprids, followed by C_4_ plants, freshwater and marine resources, while poultry and cattle are estimated to only be consumed in very small amounts, if at all. Overall, these observations align with zooarchaeological and archaeobotanical studies from *Albintimilium*^63,64^ and closeby regions^65–69^, which are discussed in more detail later.

The models also allowed us to further investigate the observed difference in δ^13^C_collagen_ and δ^15^N_collagen_ values between female and male individuals from the main necropolis phase. Both the 5-source model and the 8-source model suggest a slightly lower protein contribution in the diet of female individuals than in male individuals, and higher contributions of carbohydrates and lipids in female diets compared to male diets.

The models suggest a primary reliance on terrestrial C_3_ food sources for individuals of both biological sexes. However, it is also observable that contributions from food sources deriving from aquatic environments or a C_4_ environment are slightly higher for male individuals than for female individuals (Supplementary Fig. S17c–f).

### Strontium isotopes

#### Bioavailable strontium isotopic baseline for the Ligurian Coast

**Fig. 5:**
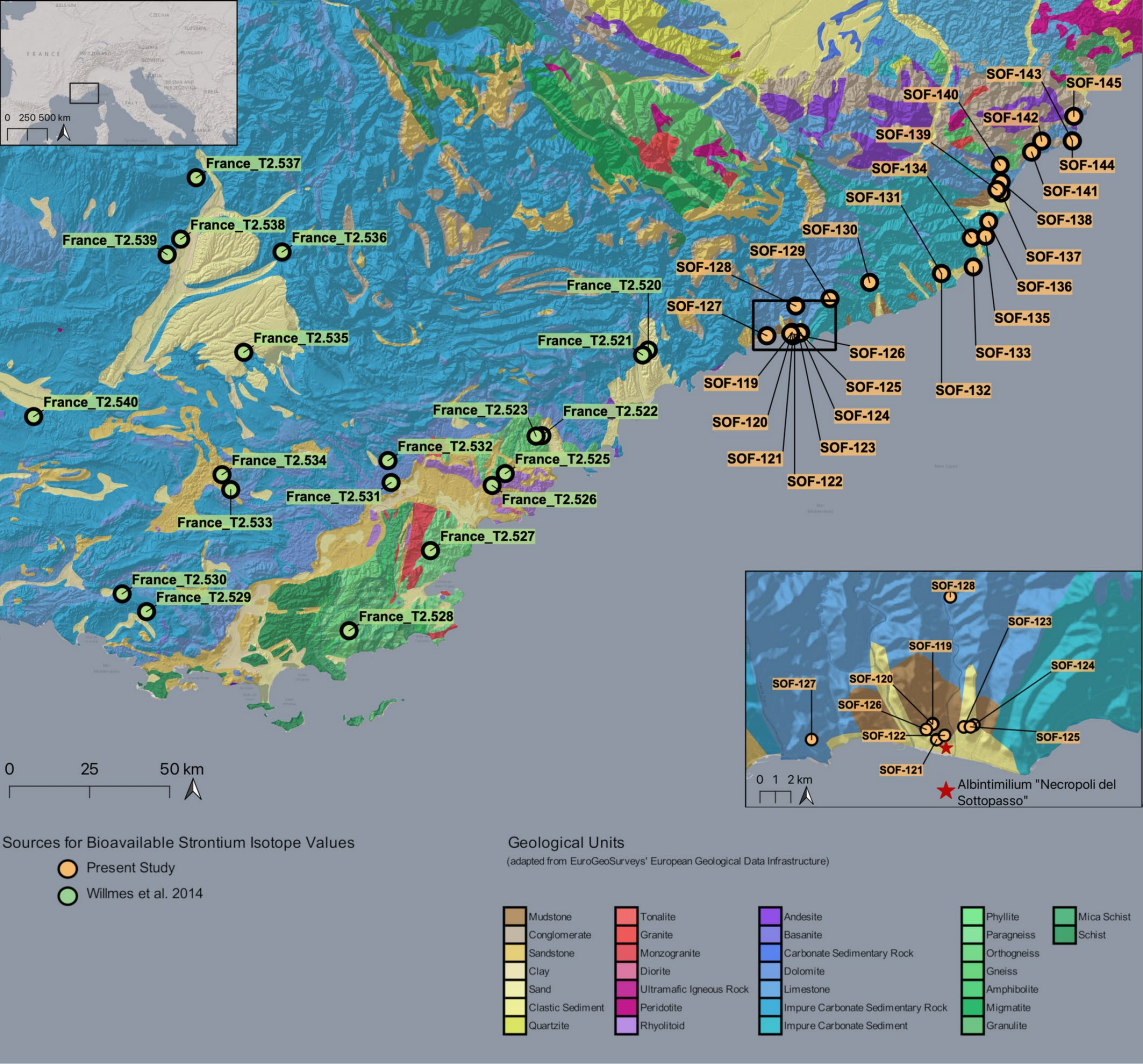
Geological map of Western Liguria (IT) and Eastern Provence (FR) showing all sampling spots used for the bioavailable strontium isoscape map. Map created on QGIS 3.16^133^ using EGDI 1:1 Million pan-European Surface Geology^134^. Basemaps: ESRI Gray (light) and ESRI Shaded Relief) © 2014 ESRI.

The geology of the Ligurian coast is very variable (Fig. 5). Detailed background ^86^Sr/^87^Sr data were not available for the area around modern-day Ventimiglia. Therefore, a variety of plant samples were gathered around the site and from the wider region to produce a bioavailable strontium isoscape map.

Bioavailable strontium sampling in Liguria yielded ^87^Sr/^86^Sr values ranging from 0.707998 in the most western sampling spot of Mortola Inferiore (SOF-127) to 0.710479 in the most eastern sampling spot close to Spotorno (SOF-145) (Supplementary Dataset S3).

We combined the data to create three ranges: The local centre, relying on the closest samples around the site (SOF-119 to SOF-126) representing the immediate environment of *Albintimilium*, an extended local centre including the closest valleys (SOF-119 to SOF-128), and an extended regional baseline including all newly gathered values of the Ligurian coast (SOF-119 to SOF-145).

These newly gathered data were subsequently combined with data from southeastern France published as part of the IRHUM database^70^ ranging from 0.707578 to 0.722785 (see Supplementary Table S6), to create an isoscape map (Fig. 7a) utilising the Bayesian geo-statistical model AverageR, which shows the modelled distribution of bioavailable strontium values in the wider region. The study area was intentionally limited to ensure that we only used data derived from plant samples collected within a similar radius and with similar coverage as the newly gathered data from Liguria. We excluded ^87^Sr/^86^Sr values from other materials, such as rocks, soils, and water (available e.g. from Lugli and colleagues^71^), to maintain consistency in the isoscape map. It’s important to note that the presented ^87^Sr/^86^Sr values are not exclusive to this region and can also be found in other areas of Europe.

#### Human ^87^Sr/^86^Sr values

**Fig. 6:**
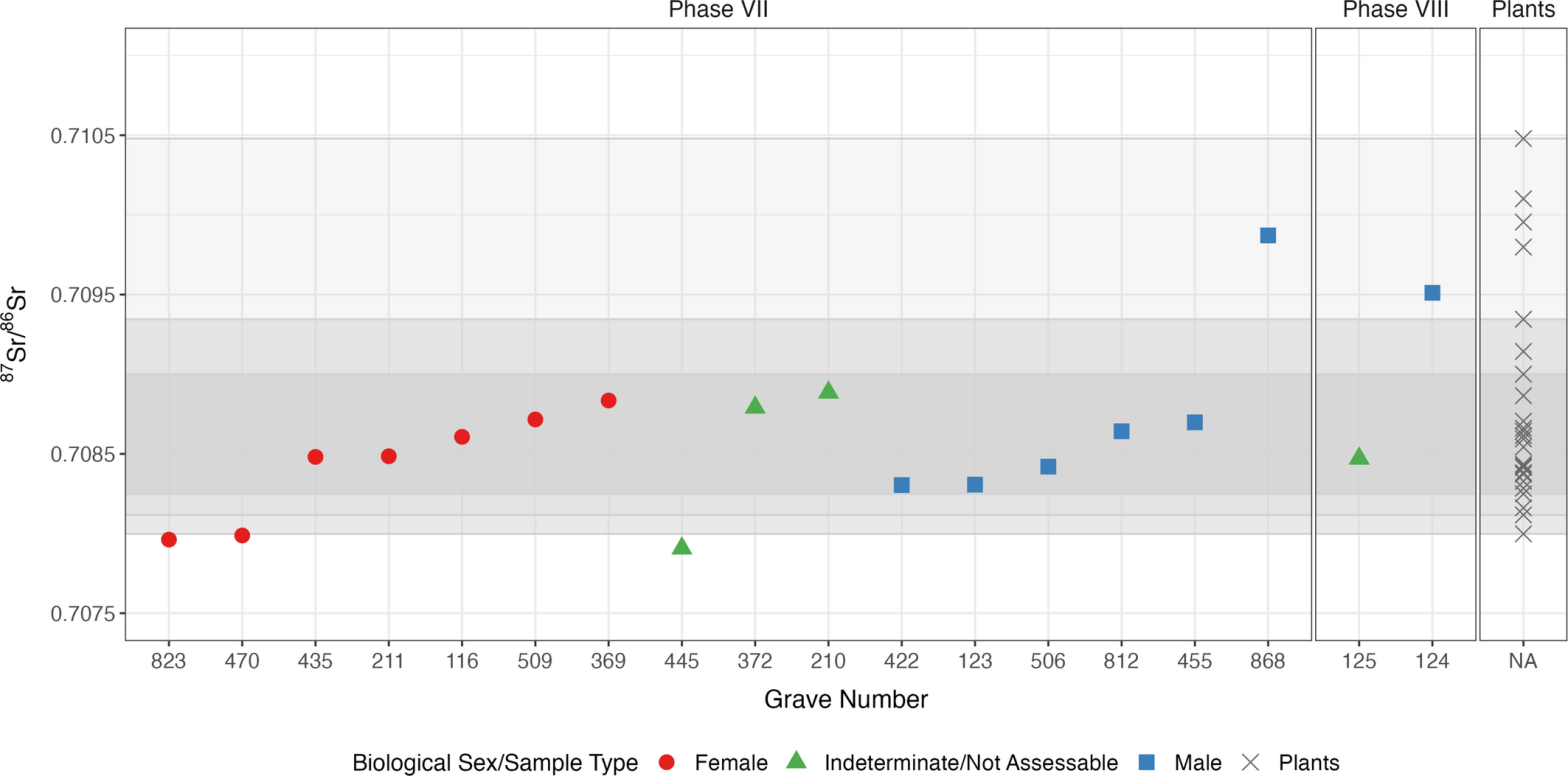
^87^Sr/^86^Sr values of humans from *Albintimilium*. Individuals are split into the two distinct burial phases and compared to the newly gathered environmental samples from Liguria (the darkest band represents the area directly around the site, one shade lighter represents the environment including samples from locations up to 10 km, the lightest band represents the wider environment along the Ligurian coast). A plot with the extended bioavailable ^87^Sr/^86^Sr ranges, including the French values from Willmes and colleagues^70^, can be found in Supplementary Fig. S18.

The ^87^Sr/^86^Sr values for the individuals from the “Necropoli del Sottopasso” of *Albintimilium* range from 0.707907 to 0.709871 and can be found in Supplementary Dataset S1. They were compared to the bioavailable baselines outlined above (Fig. 6 and Supplementary Fig. S18).

Most of the individuals from Phase VII (75%, *n* = 12/16) exhibit values which correspond to the bioavailable strontium values around the site. One individual had values which were higher than the local centre but comparable to sampling spots further down the Ligurian coast. The remaining three individuals had ^87^Sr/^86^Sr values, which were on the lower limit of the bioavailable baseline of Ventimiglia and the Ligurian coast. These, however, were comparable to values from the South of France. One of these non-local individuals was buried in a lead sarcophagus (470).

We compared the datasets of males and females but found no significant difference between them (Mann Whitney U test, *p* = 0.84).

The values of the individuals from Phase VIII are 0.708471 and 0.709511, meaning one individual (125) had a local value, while the other (124) presented a value outside the local centre but still inside the region.

**Fig. 7:**
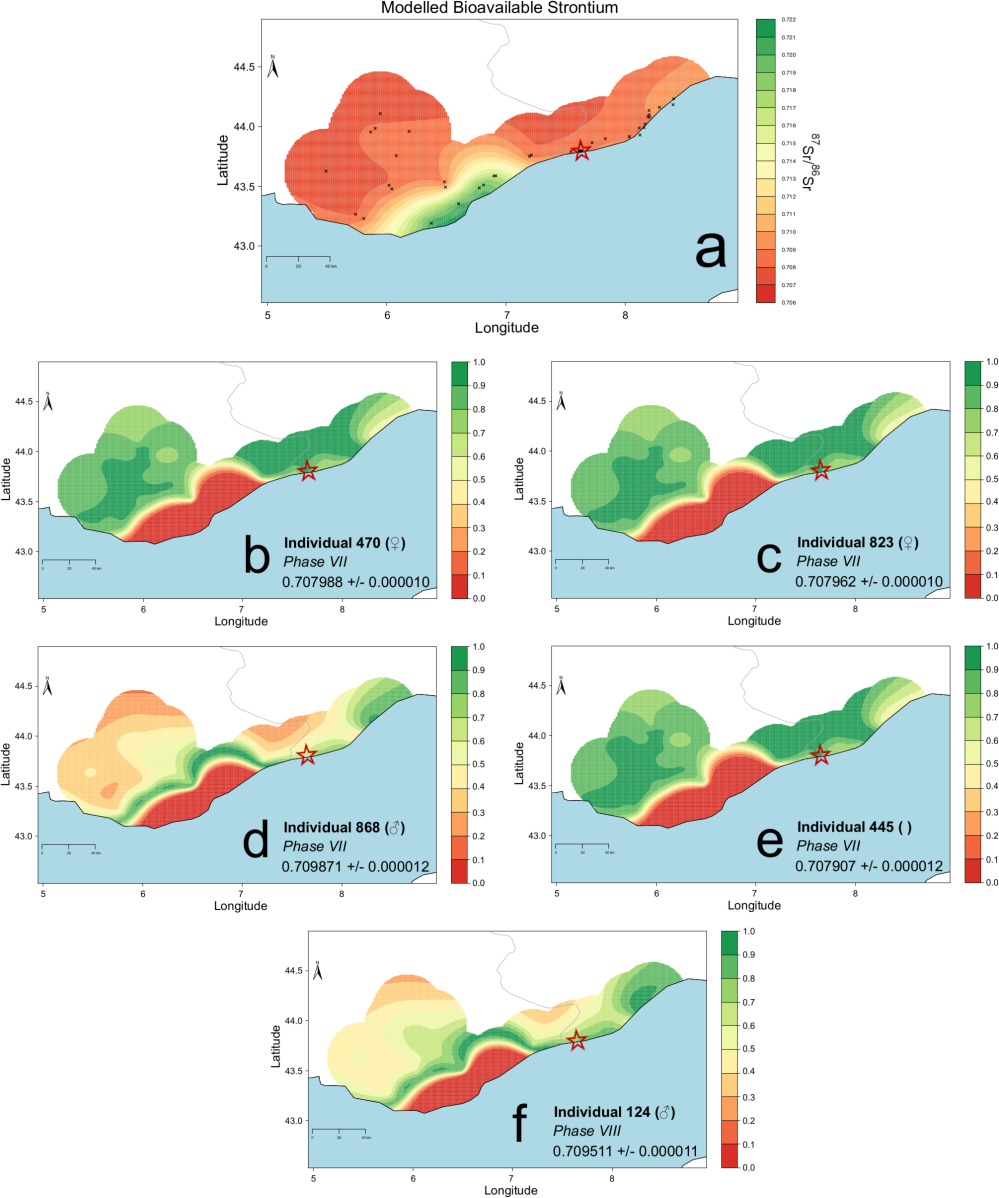
(**a**–**f**) Modelled Isoscape Map using the model AverageR and Residence probability maps using the mapping tool LocateR. (**a**) Bayesian modelled ^87^Sr/^86^Sr isoscape of Liguria and southern France (AverageR). (**b**) Individual 470, female, Phase VII; (**c**) Individual 823, female, Phase VII; (**d**) Individual 868, male, Phase VII; (**e**) Individual 445, adolescent, Phase VII; (**f**) Individual 124, male, Phase VIII; Maps were generated using AverageR and LocateR modelling options from the DSSM app (https://pandoraapp.earth/app/dssm, Spatiotemporal modelling V. 24.10.1). Model parameters are given in Supplementary Tables S7–S8. The location of Albintimilium is indicated by a red star.

Through the use of LocateR, a mapping tool that allows for the assignment of a spatial probability distribution for the place of residence of selected individuals by comparing human ^87^Sr/^86^Sr values with the spatial distribution of bioavailable ^87^Sr/^86^Sr values^62,72^, we were able to narrow down the probable (nearby) origins of the five non-local individuals from both phases within the sampled area (Fig. 7b–f), not excluding potential origin from areas further away with similar strontium isotopic values. For the three individuals with lower strontium isotopic values (445, 470, 823), most probable origins included different areas in the southeast of France, historically Southern Gaul, or the close hinterland of the site. Meanwhile, for individuals with higher strontium isotopic values (868, 124), probable nearby origins also included the Ligurian coast towards Genoa.

Comparing the values to the burial types and funerary offerings, we were able to note that individuals buried in simple pits had higher ^86^Sr/^87^Sr values, thus comparable with the Ligurian coast, while individuals buried in slightly more elaborate graves, such as the lead sarcophagus (rare in Ligurian contexts, but particularly attested for Gaul since the end of the 2nd century CE^73^), brick structure or coffins within tileboxes, showed lower values, more comparable with values in the South of France (Supplementary Fig. S19).

Overall, there is no evidence of long-distance migrations among the individuals from *Albintimilium*. All the individuals could be born either in Ventimiglia or in its neighbourhood (up to 20–30 km).

There were no significant correlations noted between dietary isotopes and ^87^Sr/^86^Sr in either the entire population (δ^13^C_collagen_ and ^87^/^86^Sr: *r*_*S*_ = -0.11, *p* = 0.68 and δ^15^N_collagen_ and ^87^/^86^Sr: *r*_*S*_=0.13, *p* = 0.63) or individuals attributed to Phase VII (δ^13^C_collagen_ and ^87^/^86^Sr: *r*_*S*_=0, *p* = 1; and δ^15^N_collagen_ and ^87^/^86^Sr: *r*_*S*_=0.27, *p* = 0.36) (see Supplementary Figs. S20–S21).

## Discussion

### Literary descriptions and dietary realities

Most of the few available historical sources portraying Liguria’s inhabitants derive from much earlier times (Diodorus of Sicily, 1st century BCE, and Strabos’ *Geography*, 1st century CE) where Ligurians, although already included in the Roman Empire, are described as still living a rather “primitive” life (Diod. *Biblioteca Historica*, 5.39.6), with occasional engagement in trade with the “civilised world” to obtain goods they cannot produce themselves (oil and Roman wine) (Strab. 4.6.2-3). These writings describe the Ligurian landscape as very harsh (*“[.] a land which is stony and altogether wretched [.]”*, Diod. *Biblioteca Historica*, 5.39.1), necessitating the reliance on pastoralism (“[.] *Ligurians*, *principally subsisting on the produce of their herds*,* and milk*,* and a drink made of barley* [.]”, Strab. 4.6.2) due to the lack of agriculturally fertile land and therefore a lack of plant-based foods (“*[.] compensate for the lack of fruits of the field by hunting game[.]”*, Diod. *Biblioteca Historica*, 5.39.3), and *“[.] eat the flesh of both domestic and wild animals[.]*”, Diod. *Biblioteca Historica*, 5.39.4). Although dietary and mobility patterns are described there, these accounts are reminiscent of the descriptions provided by various authors throughout Greek and Roman literature, who exploit the dichotomy between civilised, sedentary farmers living off domesticated plants and uncivilised, primitive herders who are “eaters of meat and drinkers of milk”^74,75^ (e.g., Herodotus about the Scythian nomads (Hdt. 4.2, 4.61), Strabo about the Gauls (Strab. 4.3), Posidonius about the Germans (Athen. 4.153e) and Caesar about Germans and Britons (Caes. B. Gall 4.1, 5.14)).

Liguria’s short valleys are ideal for transhumance, enabling flocks to be moved quickly between coastal winter pastures and higher summer pastures along routes unchanged for centuries^76–78^. Despite this, the region also provides limited but fertile land for plant cultivation, not only in the flat coastal areas but also in terraced fields, a practice evidenced in Liguria as far back as the Bronze Age^79,80^. In fact, studies on various modern European countries^81^ have shown that cereals, due to their production efficiency, can significantly contribute to caloric availability while occupying a relatively small share of land^82^. This implies that even with the area’s limited fertile land, cereals could have substantially contributed to the inhabitants’ caloric intake, therefore making the inclusion of a substantial amount of plant-based foods into the diet very feasible. This is consistent with 19th -century agricultural accounts of Ventimiglia, which describe a diet primarily based on C_3_ plants with only occasional portions of animal protein^83^.

Our results, which suggest the consumption of a mixed diet almost exclusively derived from a C_3_ environment, including variable amounts of animal proteins of different origins, agree with these observations and also match general patterns observed in other contemporary sites in the wider region (e.g., in Provence^15,16^, Late Antique Tuscany^17^, Modena^14^ and Rome^84^). Based on dental data, the whole community relied on large proportions of carbohydrates, while animal protein was consumed in varying amounts. Isotopic data indicate that the main source of animal protein was pork with consumed amounts varying within the population. Taken together, this follows the traditional idea of a heavily C_3_-based Roman economy, in which meat, when consumed, was mainly derived from omnivorous pigs^85–88^. Although the consumption of ovicaprids, a practice with pre-Roman roots, and cattle cannot be fully excluded, pigs remain the primary source of meat. Additionally, some individual isotopic variability was observed, especially in males, with some individuals (e.g., Individual 812) supplementing this diet with the occasional consumption of aquatic, most likely marine, resources, which has also been observed e.g. in Provence^15,16^. Higher amounts of terrestrial animal protein likely characterised the diet of Individual 506. Compared to other contemporary Italian sites, e.g. further up north (e.g., in the Lamon region^18^ or in Bergamo^14^), towards the east (e.g., in Friuli Venezia^89^) or in Rome^84^, C_4_ plants—commonly regarded as fallback crops^17,84,90^ associated with greater resilience to Alpine climates^89,91^—appeared to play an insignificant role in the diet of the population in *Albintimilium*.

Archaeobotanical and palynological evidence from several different archaeological contexts in *Albintimilium* dated between the 2nd and 5th century CE^57,73^, as well as from the slightly later 6th/7th CE century settlement layers at Cavo Hill and other Late Roman contexts in coastal Liguria^9,92^, give further support to this observation. They not only indicate the local cultivation of a variety of C_3_ plants (e.g. wheat, barley, emmer, various herbs, legumes like beans, vetches and lentils), fruits and nuts, and to a much lesser extent also C_4_ plants such as millets, but also suggest extensive agri- and arboricultural exploitation of the land in the region. Some sites (e.g., *Vada Sabatia*^93^) suggest little change in agricultural tradition between the Roman conquest and the following centuries. Evidence for the production of other foodstuffs, including wine, is scarce in Late Roman Liguria, but they were seemingly imported through the trade network which connected the region with North Africa and the Eastern Mediterranean, as evidenced by the presence of imported amphorae^20,21^.

Zooarchaeological evidence from various contexts in *Albintimilum*^63,64^ dated between the 2nd century BCE and the 5th century CE, as well as from other Late Roman sites in the region^65–69^, highlights a significant reliance on pork. This is reflected in the consistently higher quantities of pig bones (up to 51% of the studied assemblages^63,64^) compared to those of other taxa.

Overall, these records align with resources typically associated with a Mediterranean Roman diet, where the common dietary intake of a Roman population predominantly consisted of bread made from C_3_ plants such as wheat and barley, along with grain-based porridges. Additionally, vegetables, fruits, pulses, and sporadic servings of animal protein in the form of dairy, (terrestrial) meat, or fish would have also been included^75^.

Our results thus falsify the Greek and Roman topos (literary pattern) of presenting people living in more distant or marginal Mediterranean environments as barbarians incapable of subsisting on agriculture. This is further emphasised by the available comparative studies, which show that the same “Roman” diet, with minor variations in crop preference, was practiced across a wide range of environments, from the coastal mountain regions of Provence^15,16^ and Liguria to the Alps^14,18^ and the lowlands of Northern Italy^17,89^. The community buried in *Albintimilium* was part of a heavily Roman-influenced society, living in a Roman city with established trade connections to the capital and other parts of the Empire, as evidenced by the archaeological context, including burial types and grave goods.

While we are first to point out the discrepancy between literary depictions and actual diets of more distant or marginal Mediterranean populations and the first to use isotopic data to do it, the unreliability of dietary information coming from literary sources has been demonstrated also for mediaeval Anatolia with regard to the type of bread and flour consumed by different socio-economic groups based on the study of dental enamel wear^94^.

### Aquatic resources in the diet

Individuals from *Albintimilium* may have had access to aquatic resources due to the site’s coastal location and its position between two rivers. These bodies of water support various marine, freshwater and catadromous fish species, as well as molluscs, which are still present today. Zooarchaeological studies from *Albintimilium* also report the presence of several molluscs with cut marks as well as very small amounts of fish bones. Molluscs are also reported in other nearby sites (e.g., Arles^68^). A potential fishhook found among the artefacts in *Albintimilium*, along with ample evidence of fishing activities in other contemporaneous coastal towns in the region (e.g. Noli^95^ and Castrum Perti^96^), also support the inclusion of resources from both freshwater and marine environments in the diet. However, the resource estimation for this community shows a stronger reliance on foodstuffs from C_3_ environments, and particularly C_3_ plants (41–98‰), with much lower contributions from aquatic or C_4_ environments. The high levels of carious lesions in the community, and particularly in females, give further support to a carbohydrate-rich diet. Our results, therefore, advocate for the consumption of only small amounts of aquatic, particularly marine, resources within this community as a whole, although some individuals with higher δ^13^C_collagen_ and δ^15^N_collagen_ values may have included slightly higher proportions of these. This surprisingly overall weak signal from freshwater and marine resources, particularly considering the site’s location, has also been noted in other isotopic studies from various regions of the Mediterranean^17,97,98^.

### Diet and connectedness

Although these general patterns support the idea of a population following a traditional Roman diet, the question remains as to what degree this community was connected to other parts of the Empire.

The presence of imported vessels, often associated with the oil and wine trade, suggests that individuals from *Albintimilium* were able to rely on the supply network of the Roman Empire to some extent. However, our results indicate that inhabitants of *Albintimilium* primarily relied on local resources that could be found within a radius of approximately 30 km from the site, probably sourced through a network of smaller farms and villas. Farms like this are not only mentioned in written sources (*Tac. Ag. 7* mentions a *praediis* (farm estate) in *Intimilium* (the area around *Albintimilium*^[Kraus 99^)), but have also been attested archaeologically close to *Albintimilium*^100^.

Overall, we found no evidence for long-distance migration or the consumption of large amounts of imported foodstuffs, similar to the results from Lamon^18^. However, the data suggest that regional mobility played a relatively important role, indicating that the community was not completely isolated but included individuals from other areas within the wider region and had occasional access to imported goods. This could have particularly been the case for individuals diverging from the general pattern as evidenced by their ^87^Sr/^86^Sr values.

We found no significant difference in strontium isotopic values between sexes. The majority of both males and females show values that are consistent with the local environment. However, we were able to observe that some women showed values which were typical for Southern France, while some males showed values more expected along the Ligurian coast. The female individuals with strontium isotopic values outside the local centre were amongst the individuals buried in slightly more elaborate graves (470 in a lead sarcophagus, 823 in a coffin surrounded by stones within a tiled box) and had evidence of funerary goods or special treatment (470 floral bouquet and pillow, 823 knife, headpins and a clay lamp). This suggests that some female individuals with higher social status, as indicated by their burial type, joined the community from different areas within the same region, potentially hinting at exogamy.

The presented evidence suggests that individuals from *Albintimilium*, regardless of sex, adhered to a diet heavily reliant on a limited set of local resources, with only limited input of potentially luxury imported goods (e.g., wine).

The presence of a few outliers suggests some degree of individual variability or preference for certain (potentially imported) resources in the diet, or possible non-local origin that, in some cases, may be connected to exogamic practices.

### Dietary differences between sexes

Importantly, we did observe some differences in diet within the community. These could not be associated with differential burial treatment, such as privileged burial types or inclusion of grave goods.

Although there is a larger variety in burial types and grave goods in female burials compared to a more uniform burial rite for males, the opposite pattern is observable in their diet. Isotopic values of female individuals exhibit lesser dispersion compared to those of male individuals, indicating more uniform dietary habits of females. The significant difference between female and male individuals in both δ^13^C_collagen_ and δ^15^N_collagen_ values, along with the higher prevalence of dental pathologies in females, suggest markedly distinct long-term diets between the biological sexes.

In a traditional Roman society, women primarily handled domestic tasks, while men, if economically possible, took on public or military roles. These social distinctions influenced the distribution of dietary resources and overall dietary patterns within families^24,75^. Food distribution and consumption, adhering to societal hierarchies, can create divisions within the community by reflecting existential, cultural, social, and economic dimensions. Within families, food allocation often indicated relative power and status, affecting dynamics between parents and children, different age groups, and genders^75^.

This finds further support in ideas on the health of women and their dietary needs as bequeathed through medical writings of the intellectual elites (e.g., Athenaeus of Attaleia in *Orib. Lib. Incert.* 21, or Rufus of Ephesus in *Orib. Lib. Incert.* 20), in which fish and some meats are not recommended for women. Although these writings cannot be treated as a representation of lived ideals of individuals, they may still be used to get a general idea of an existing imbalance in the distribution of resources rooted in underlying societal structures.

Due to the vast size and duration of the Roman Empire, as well as potential variations in personal choices among individuals and communities, which are nearly impossible to trace archaeologically, it is unlikely that there was a singular, universal method of food distribution among communities based solely on gender.

This diversity is evident in the varying results of previous bioarchaeological studies, which have focused either on the prevalence of specific oral pathologies as markers for dietary composition or on the analysis of stable isotopes.

These gendered differences are visible mainly in Italian case studies (e.g., Hellenistic-Early Roman Sicily^26^, Imperial Isola Sacra^24,86^, Early Imperial Velia^101^, and Herculaneum^102^), potentially suggesting that sites in closer geographical proximity and area of influence to the political and cultural centre of Rome show greater differences between sexes and potentially higher degree of adaptation of a Roman lifestyle than sites located outside the core area of the empire (e.g., 1st -4th century Macedonia^103^, 1st -4th century Lebanon^25^, or 3rd -7th century Southern Gaul^15^). However, to investigate this hypothesis further, more studies are needed.

Based on the isotopic and dental data, female individuals in *Albintimilium* appear to have consumed smaller proportions of animal proteins, similar to the individuals from Isola Sacra^24,104^.

The strong positive correlation between δ^13^C_collagen_ and δ^15^N_collagen_ values suggests that women had variable access to animal protein. However, it is unlikely that this protein was derived from animals with higher isotopic values, such as marine resources. Although this may be associated with a deliberate choice made by women due to physiological differences between sexes, it also aligns with the ideas portrayed in medical writings mentioned earlier.

The smaller ranges of both δ^13^C_collagen_ and δ^15^N_collagen_ values in female individuals additionally hint at less variability and potentially more reliance on cereals and legumes by individuals with low δ^15^N_collagen_ values. The significantly higher number of carious lesions in female individuals gives further support to this observation.

Male individuals, however, show isotopic values consistent with a more varied diet with a larger share of animal products and the occasional inclusion of foods with higher isotopic values, such as marine fish, animal products from omnivore or infant animals, or foodstuff of foreign origin with elevated baseline δ^13^C and δ^15^N values, such as those sourced from trading or hunting. Their significantly higher number of teeth affected by calculus may also be used as an indicator for a diet richer in protein, although calculus is known to have a multifactorial aetiology.

While, the community’s overall dietary pattern, coupled with the absence of long-distance migration, suggests a localist community with limited movement of people and minimal reliance on (long-distance) imports, the differential treatment or access to dietary resources, particularly those with higher protein contents, suggests that imperial Roman societal values, norms, and ideas were adopted, nonetheless.

### A glimpse into the troubled 5th century CE?

The isotopic and osteological results obtained from two individuals attributed to the last phase of the necropolis hint at a potential change in living conditions and diet accompanying the gradual abandonment of the Roman city. However, with isotopic data from only two individuals, these conclusions remain tentative. The remains of all four individuals attributed to this phase were buried in simple pits, which were placed into a sand layer associated with the gradual abandonment of this part of the Roman city. These individuals were buried some time in the second half of the 4th or the beginning of the 5th century (see Supplementary Note S5 and Supplementary Figs. S22-S24 for detailed ^14^C results), without any grave goods. Only two of these individuals could be analysed in detail.

These two individuals had the lowest isotopic values in both δ^13^C_collagen_ and δ^15^N_collagen_ amongst the entire sample. This suggests a more limited intake of animal foodstuffs, likely substituted by a diet richer in cereals and legumes. There is no evidence for the direct consumption of C_4_ plants (e.g., millets) or marine resources among these individuals. Their ^87^Sr/^86^Sr results do not provide insights into changes in mobility patterns; one individual exhibited values consistent with the site, while the other had non-local values from the wider region.

The 4th and particularly the 5th century were marked by intensive military activity in Northern Italy, primarily caused by Gothic invasions under Alaric I (395–410 CE) and his successors, most likely leading to increased instability and insecurity. The refortification of the nearby town of *Albingaunum* (modern Albenga) by Constantius between 410 and 420 CE might be used as indirect evidence that instability also affected Liguria^9^.

The individuals attributed to the later phase may be used to gain insight into the living conditions during this period. Overall, these data could hint at a change in dietary patterns with lesser consumption of animal proteins.

## Conclusions

The present study is the first of its kind in this part of the Italian Peninsula during the Late Roman period and, therefore, provides valuable insights into a hitherto under-researched part of the Roman Empire. Our data point to a localist population, which was autonomous to a great extent. Contrary to the perceptions shaped by literary sources, our data show that both overall dietary patterns and sex-based dietary differences within this community display a strong Roman character.

However, despite the population’s “Roman” diet and social structures, there was a surprising lack of interconnectedness regarding human mobility and food distribution. This suggests that, while cultural practices like general dietary patterns may have aligned with Roman norms, the main infrastructure of food distribution and resource exchange remained local, with *Albintimilium* mostly relying on a network of surrounding farms and villas.

Although *Albintimilium* was situated on a popular commercial route, which could have facilitated the movement of people and goods, the population of Late Roman *Albintimilium* showed little evidence of extensive migrations or significant input from foreign produce; while pottery evidence indicates the presence of imported, locally unavailable goods (e.g. wine, oil, fish sauces), they likely were not widespread enough to have a noticeable impact on the isotopic values of individuals. Whilst food was being transported, large quantities were likely to primarily reach specific destinations such as military camps and large metropoleis like Rome and Constantinople, which could not rely solely on the production of their surrounding countryside^105^.

The sample size and the local nature of the data restrict the findings to the specific region under study, making it difficult to extrapolate them to the diverse expanse of the Roman Empire. Nonetheless, this research provides a fresh impetus for a more nuanced understanding of daily life, the use of local and non-local resources, and the role of intra-regional trade networks. This perspective, which requires further investigation, suggests that people in the Roman Empire might not have depended or relied entirely on widespread resource sharing between regions for their sustenance. Instead, they appeared to engage more deeply with localised and localist practices and traditions, diverging from the unified economic and cultural Mediterranean *koine* imposed by Roman rule.

## Methods

### Osteological Analysis

Biological sex of adult individuals was assessed using morphological traits in the pelvic girdle^106–108^, skull and mandible^109,110^, with a preference for pelvic data, if available, and post-cranial metrics^111^. Sex estimation was not attempted for non-adult individuals.

Estimation of age-at-death in adults relied on evaluating the morphology of the pubic symphysis^112,113^ and auricular surface of the illium^114^, resorting to dental attrition^115^ when necessary.

For non-adult individuals, age-at-death estimation primarily relied on dental formation and eruption status^116–118^, which was supplemented by the assessment of epiphyseal fusion^119^ and measurements of long bones^120,121^. Additionally, cranial bone measurements were used for pre-term/foetal and neonate individuals^119,122^.

Individuals were then assigned to one of nine age-at-death categories following Buikstra and Ubelaker^106^ and Scheuer and Black^123^ (Supplementary Table S2). Methods are described in detail in Supplementary Note S2.

Carious lesions were recorded after Lukacs^124^ per anatomical position on the tooth surface (occlusal, lingual, buccal, medial, distal, interproximal, multiple) and severity (pit/small fissure, medium-large, large, only root remaining), and dental calculus was recorded after Brothwell^115^ per tooth and severity (slight, medium, considerable).

In order to maximise sample size for statistical analysis, all recorded dental conditions were converted to presence/absence scores per tooth.

### Isotope analysis

#### Carbon and nitrogen

To produce insights into the diet approximately five years prior to the death of an individual^35,125^, we sampled rib fragments due to their relatively fast bone turnover rate. To provide a baseline for the interpretation of the 18 human samples, we additionally sampled remains from 19 animals deriving from various stratigraphic units from the same excavation, starting at the foundation of the republican walls (1st century BCE), all the way to the necropolis horizon.

Collagen was extracted from human and animal bones using the established laboratory procedure of the Department of Bioarchaeology at the University of Warsaw following the protocol by Longin^126^ with modifications. Bone fragments, ranging from 400 mg to 600 mg, underwent manual abrasion, followed by demineralisation in 0.3 M HCl water solution at room temperature until the mineral components were dissolved. Subsequently, the samples were washed with deionised water, gelatinised in hydrochloric acid solution (pH 3) at 70 °C for 48 h, filtered using Ezee Filter separators, and then lyophilised.

Extracted collagen was analysed at the Vilnius Radiocarbon Laboratory using an Elementary Isoprime Vision Mass spectrometer connected to a Vario Isotope Cube elemental analyser. Measurement of elemental concentration was standardised based on acetanilide. Samples were measured against internal (acetanilide) and international (USGS40 and USGS41a) standard materials. Measurement error, expressed as 1 sigma, was estimated based on the repeated measurements of internal standard material (better than ± 0.2‰ for δ^13^C and ± 0.3‰ for δ^15^N).

For further analysis, samples with carbon concentration above 13%, nitrogen concentration above 4.8% and atomic C/N ratio between 2.9 and 3.6 were accepted^58–60^.

To support the archaeological dating, collagen samples of two individuals (125 and 455), representing both phases, were also radiocarbon dated at the Vilnius Radiocarbon Laboratory using Low-Energy Accelerator (LEA, Ionplus AG, Zürich) and Automated Graphitization Equipment AGE-3 (Ionplus AG, Zürich). NIST-OXII and phthalic anhydride were used as reference materials. The ^14^C/^12^C ratio was measured with an accuracy better than 0.3% (+/-30 years or less), and the ^13^C/^12^C ratio was used for the correction of isotopic fractionation. The dates were modelled using the IntCal20 calibration curve^127^.

#### Strontium

In order to reflect early childhood residency, we sampled enamel from early developing teeth, such as first molars or central incisors. Depending on the crown preservation, this was either done through collecting pieces of enamel or through direct extraction of enamel powder (up to 20 mg) perpendicularly to the growth axis of the tooth with a handheld drill at the aDNA laboratory of the Masaryk University in Brno.

Homogenisation of plant samples, chemical separation of Sr and measurements of Sr isotope ratios were carried out at the Isotope Laboratory of the Adam Mickiewicz University in Poznań. Tooth enamel samples were cleaned in an ultrasonic bath with deionised water to remove sediment particles. Afterwards, 11–13 mg of powdered enamel was treated sequentially according to the procedure described by Dufour and colleagues^128^ with 0.1 ultrapure acetic acid (5 times) to eliminate the diagenetic Sr contamination. Subsequently, the enamel samples were dissolved on a hot plate (~ 100 °C, overnight) in closed PFA vials using 1 N HNO_3_. The powdered plant samples (~ 90–100 mg) were dissolved on a hot plate (~ 100 °C, 3 days) in closed PFA vials using a mixture of concentrated hydrofluoric and nitric acid (4:1).

The miniaturised chromatographic technique described by Pin and colleagues^129^ was applied for Sr separation, with some modifications in the column size and concentration of reagents^130^. Strontium was loaded with a TaCl_5_ activator on a single Re filament and analysed in dynamic collection mode on a Finnigan MAT 261 mass spectrometer. Total procedure blanks were less than 80 pg. The ^87^Sr/^86^Sr values were corrected to ^86^Sr/^88^Sr=0.1194. The Sr results were normalised to the certified value of NBS-987 = 0.710340.

More detailed descriptions of laboratory procedures for carbon and nitrogen isotopic analyses can be found in Supplementary Note S3.

### Statistical analysis

Statistical testing was conducted to identify significant differences in stable and radiogenic isotope values between subsets of different biological sex.

As the normal distribution cannot be expected in the isotopic dataset, only non-parametric statistical tests were performed, including the Mann-Whitney U-test for two groups. Spearman’s correlation coefficient was calculated for δ^13^C_collagen_, δ^15^N_collagen_ and ^87^Sr/^86^Sr values.

Chi-square tests were used to examine the association between dental pathologies and biological sex.

Statistical testing was applied only when the size of every compared subset was higher than five individuals.

Due to the small sample size, no further statistical tests exploring differences based on age-at-death or targeting socio-economic differences based on grave goods or burial types could be undertaken.

Excluding individuals 124 and 125 from inter-population comparisons was deemed necessary due to their association with the latest phase of the necropolis. The inclusion of these individuals, characterised by distinct chronologies, would introduce potential confounding factors and compromise the clarity of the analyses. Due to the small sample size, no statistical analyses could be undertaken comparing the two phases. However, some qualitative observations are reported.

All statistical analyses were performed using R Statistical Software (v4.3.0) with the *stats*^131^ package, and the significance level was set at *p* < 0.05.

#### Bayesian modelling

All three applications discussed in the following are included in an R-based^131^ Open Source package developed within the Pandora & IsoMemo initiatives (https://www.isomemoapp.com/*).*

#### ReSources

To obtain quantitative dietary contributions, the δ^13^C and δ^15^N values were used in the Bayesian dietary mixing model ReSources. ReSources allows for the quantification of both caloric and macronutrient (protein, carbohydrates/lipids) contributions from different food sources that make up an individual’s diet. Model details can be found in Supplementary Note S4 and Supplementary Tables S3–S5.

#### AverageR

With the ^87^Sr/^86^Sr values from environmental samples, we created a Bayesian spatial ^87^Sr/^86^Sr isoscape using the Bayesian geo-statistical model AverageR. AverageR estimates the expected value of a dependent variable (in this case ^87^Sr/^86^Sr value) across space (for method details, see^62,72,132^). Modelling parameters are specified in Supplementary Table S7.

#### LocateR

We used this Bayesian reference baseline obtained through AverageR to further identify the most probable place of origin of individuals with non-local but regional signatures, using the mapping tool LocateR (for method details, see^62,72,132^). Modelling parameters are specified in Supplementary Table S8.

## Electronic supplementary material

Below is the link to the electronic supplementary material.

Supplementary Material 1

Supplementary Material 2

Supplementary Material 3

Supplementary Material 4

## Data Availability

Data supporting the findings of this study are available in the Supplementary Information file and Supplementary Datasets S1-S3.
