# Supplementary material for: Isotopic data reveal a localist Roman population in late Roman Albintimilium, Liguria
Source: Sci Rep. 2025 Apr 9;15:12097. doi: 10.1038/s41598-025-92851-7 (PMC11982215; doi:10.1038/s41598-025-92851-7)
Supplement: Supplementary file 4 — Supplementary Material 4 [file 41598_2025_92851_MOESM4_ESM.pdf]

Supplementary Information

Isotopic Data reveal a localist Roman population in Late Roman *Albintimilium*, Liguria

Sarah Defant*[1,2], Alessandro Carabia[3], Rafał Fetner[4], Elizabeth Craig-Atkins[5], Ricardo Fernandes[2,4], Gian Piero Martino[6], Stefano Costa[7], Arkadiusz Sołtysiak[4], Adam Izdebski*[2]

[1] Institute of Prehistoric Archaeology, Freie Universität Berlin, Fabeckstr. 23/25, 14195 Berlin,Germany
[2] Max Planck Institute of Geoanthropology, Kahlaische Straße 10, 07743 Jena, Germany
[3] School of History, Classics and Archaeology, Newcastle University, Armstrong Building, Newcastle University, Newcastle upon Tyne NE1 7RU, United Kingdom
[4] Department of Bioarchaeology, Faculty of Archaeology, University of Warsaw, ul. Krakowskie Przedmieście 26/28, 00-927 Warszawa, Poland
[5] School of History, Philosophy and Digital Humanities, The University of Sheffield, Western Bank, Sheffield S10 2TN, United Kingdom
[6] Former Soprintendenza per i Beni Archeologici della Liguria
[7] Soprintendenza Archeologia, Belle Arti e Paesaggio per le Province di Imperia e Savona , Via Balbi 10, 16126 Genoa, Italy

*Corresponding Author (sarah.defant@fu-berlin.de, izdebski@gea.mpg.de)

This PDF file includes:

Supplementary Notes S1-S5
Supplementary Figures S1 to S24
Supplementary Tables S1 to S8
Supplementary Information References

Other supporting materials for this manuscript include the following:

Supplementary Datasets S1 to S3
ReSource Models

Supplementary Notes

Supplementary Note S1: Archaeological & Historical Background

S1.1 Previous Archaeological Interventions in *Albintimilium*

Girolamo Rossi (1831-1914), a local pharmacist and amateur archaeologist, conducted some of the first documented excavations in *Albintimilium* between 1876 and 1904 when he was the local inspector of antiquities[1]. At that time, the site of Roman *Albintimilium* was mostly covered by fields, which started to be resettled due to the expansion of the medieval town on Cavo Hill back into the plains. The growing number of constructions in the area began to expose several archaeological finds, many of which were acquired by private collectors and dispersed. Rossi was able to acquire and salvage some of these materials while he excavated the Roman theatre and the baths. The Italian Government undertook more systematic excavations under the direction of Piero Barocelli (1887-1981) from 1915 to 1918. He used prisoners of war to continue the exploration of the theatre, some of the *insulae* north of it, and a vast section of the Roman necropolis, which extended along the Roman road west of the settlement, where he documented 145 cremations[2] (Supplementary Fig. S1).

Nino Lamboglia (1912-1977) was the first archaeologist to conduct a vast series of excavations in the settlement, applying a rudimentary, but innovative for the time, stratigraphic method from 1938 to his death[3,4]. These campaigns uncovered several parts of the city, plus a series of monumental funerary enclosures on the road's northern side, dating from the second quarter of the 1st century CE to the late 2nd century, to complement the finds of Barocelli on the southern part of the street. The necropolis likely extended westward for about one kilometre. Repeated excavations have exposed nearly 230 burials in this funerary area to date. This includes the first nucleus of inhumation burials on the western outskirts of the theatre, which appeared between the end of the 2nd century and remained in use until the 4th century[5]. Afterwards, the theatre was used for funerary purposes between the 5th and at least the 7th centuries. Other scattered late antique depositions have been located along the *decumanus maximus*, plus one isolated infant burial in the Roman baths[6,7]. A large cemetery preliminary dated to the 6th-7th centuries is currently under excavations outside the northern curtain wall[8].

Since Rossi's discoveries, all burials in *Albintimilium* have been numbered according to a continuous sequence, with the ones of the “Necropoli del Sottopasso” representing the numbers from 229 to 274. However, we decided to keep the original context numbers to better correspond with the original documentation produced during the excavation. The excavation was executed following a series of “interventi” (trenches or sondages) distributed across the area of interest, later unified to look like an open-area excavation (Supplementary Fig. S2).

The archaeologists excavated fifty burials, most of which were available for analysis. Four burials were identified but not excavated, making it impossible to determine the number of depositions present; one *enchytrismòs* (43 / 143) excavated by Lamboglia

in the 1970s could not be located in the deposits; another *enchytrismòs* (97/96) was removed, possibly for micro-excavation in the laboratory, and was also not available. Lastly, a series of cuts (56, 54, or 383) were interpreted as possible burials that were never used or where the body was removed in antiquity.

A structured necropolis?

The necropolis appears to be organised around three or four nuclei centred on privileged depositions. Among the earliest, if not the very first, inhumation in the area is the monumental burial 235, surrounded by a series of adult and infant burials. Shortly after, to the south of 235, two similar monumental structures (362 and 363) were erected, also attracting a group of depositions. Almost simultaneously, in the northernmost part of the graveyard, an elderly woman was buried in a lead sarcophagus (470), around which another set of burials seemed to have formed. These first "foundation" burials are all oriented east-west and are divided into two typologies: monumental structures (235, 362, and 363) and lead sarcophagus (470). The latter underwent detailed analyses directly after excavation. Archaeobotanical samples were also identified, revealing the presence of a funerary deposition of flowers alongside traces of pollen from local flora. Textile fragments were recovered, suggesting the presence of a valuable shroud for the body and a funerary pillow[9]. Thus far, including "luxury" elements such as the pillow and shroud, coupled with the ritual deposition of flowers and the distinctive burial, may indicate rites reserved for unique individuals of particular social or religious standing. Later depositions covered the sarcophagus without disturbing it, possibly suggesting a preservation of its memory.

The three monumental structures are instead separated into two groups: 235, located more or less in the centre of the necropolis, while 362 and 363, were placed almost one beside the other, south of 235. They all share the same rectangular, parallelepiped form and construction technique, with only 363 being slightly smaller than the other two. They were all constructed using a mix of local stones and fragments of ceramic building materials, forming a larger base receding into a smaller elevated structure. Traces of painted reddish plaster were found still in place at the base of all them.

The fourth and last section is located at the southernmost edge of the excavation. Here, the remains of a dry stone structure (299) demark an area possibly reserved for infant burials where four subadults (285, 293, 300, and 311), all deceased within the first twelve months of their lives, were discovered. However, this argument is weakened by similar burials found elsewhere in the necropolis. The depositions 14 and 557 gravitate towards the edges of the nucleus centred around the monumental burial 235, somehow reinforcing the argument of newborns placed on the outskirts. However, newborn 245 is positioned just beside 235, almost at the group's centre. In the nucleus marked by the lead sarcophagus (470), the infant 449 is deposited again at the centre, almost on top of the sarcophagus itself. Of eight newborns, six are placed in marginal areas, and two are placed in central positions. Statistically, the data suggest some form of separation between newborns and adults/subadults, as shown in other case studies[10], with only a few exceptions. However, our understanding in this case is limited by the partial excavation of the necropolis, which does not allow for a reconstruction of the entire plan. In this sense, the southernmost group may simply represent a portion of a larger nucleus of mixed adult-infant burials yet to be

discovered. At the same time, the drystone wall could merely be a containment wall, marking a slope change, as suggested in some of the original excavation documents.

A final mention regarding the necropolis is the presence of a well built while the site was in use as a graveyard. The well is made of stone and presents three niches at its base. On its side, the remains of an upside-down *dolium* have been recovered. The well is located between the area of the lead sarcophagus and the one of the monumental burial 235 (Fig. 1 in the Main Text), within a space relatively free of depositions. Its function, in connection with the necropolis, is unclear and will deserve more attention to see if it could have been linked to some part of the funerary rite, together with the *dolium*, which might have worked as a container of some sort.

S1.2 Finds associated with the Necropolis

The necropolis occupied an area that was previously free of structures or previous burials. According to the excavation reports, earliest phases are related to the construction of the republican walls and a series of ditches. Its proximity with the cremations and with the theatre, where other contemporary burials were identified, could have resulted in the spoliation of some previous funerary structures in the vicinity. This could be the origin of an epitaph that has been found broken in six pieces recovered during the excavation (Supplementary Fig. S3), two of which were found in burials 14 and 24. An unnamed husband dedicated the epitaph to his deceased twenty-nine-year-old wife, *Sextilia Peterna*. Stylistically, the epitaph dates to the late 2nd century and the mid-3rd[11]. It is possible that the epitaph came from the nearby cremation of the Western Necropolis, however its date can also imply a reuse within the "Necropoli del Sottopasso" itself.

Other relevant finds for our casestudy are represented by the recovery, in a few burials, of a series of funerary goods (Supplementary Table S1), only some of which we were available for this study. Four of the nine burials accompanied by grave goods are attributable to female individuals. For the other five, no definite sex could be assigned, either due to them exhibiting ambiguous features, which could be attributed to the old age of the individual (210), or them being too young to have pronounced definite sexual dimorphism (68). Two non-adults (386 and 445) exhibited some features which could be attributed to female individuals. However, due to their young age, it was decided not to assign them a biological sex. The last skeleton (43) was not available for analysis, but according to the archaeological reports, it should belong to a non-adult. As noted in the article, the data seems to show a continuity of the rite of depositing grave goods along the deceased with a marked preference for female individuals, which might denote some form of socio-cultural differentiation based on gender. In the following pages, we will describe some findings that we were able to study in association with the related burials, considering only the adults.

Burial 210

Old Adult, indeterminate (Supplementary Fig. S4). The body was deposited in a wooden box, reconstructed through the presence of six nails regularly distributed around the skeleton. It was oriented W-E (head E) with a curvilinear iron object intentionally placed in the mouth and with a clay lamp at the feet (Supplementary Fig. S5). The lamp belongs to the group of the "Firmalampen", originally produced in the

Po valley since the Flavian dynasty and then diffused in the northern region of the Empire (Gaul, Britain, and Germany).

Clay lamp imitation of “Firmalampen” X-c
Typology: imitation of Firmalampen X-c
Production Area: Local
Chronology: 3rd-4th century
This is a local copy of Firmalampen, as attested by the product's poor quality, the clay's lower level purity, and the absence of any production seal (Supplementary Fig. S5). It was made using the technique known as *surmoulage*[12]. The form is similar to a Firmalampen type X, variant c, as identified by Buchi[13]. Like most examples of this typology, it lacks an air hole, and the base is flat, missing the usual single or double ring typical of the other types and subtypes. Copies of this kind were mainly diffused during the 3rd and 4th centuries, even if, in some cases, they are attested until the 5th [14].

Burial 386

Adolescents, possibly female (Supplementary Fig. S6), but due to their young age, it was impossible to determine the sex securely. The body was deposited in a wooden box, testified by the presence of 18 nails distributed around the skeleton, with the box encased into a tile box (Supplementary Fig. S7). It was oriented N-S (head N) with a clay lamp between the feet and a small olla with a broken handle beside the right foot. The lamp was not available for study at the time of this study, while the olla (Supplementary Fig. S8) is currently under examination together with the rest of the site’s ceramic.

Burial 435

Young adult, female (Supplementary Fig. S9). The body was deposited in a tile box oriented W-E (head E); the presence of a wooden box has been suggested based on the positioning of the feet. A necklace of 31 glass beads of various forms and dimensions was deposited in the grave, together with a fragmented clay lamp. Due to the lamp's fragmentary status and the lack of conservative intervention, it is still impossible to establish a proper typology.

Burial 509

Old adult, female. The body was deposited in a wooden box, as testified by the presence of at least four iron nails and other iron fragments. The body was oriented W-E (head E) (Supplementary Fig. S10). She was buried with a clay lamp (Supplementary Fig. S11) and an olla, both intentionally broken in antiquity and placed at her feet.

Clay lamp Loeschcke VIII / Bailey Q (x)
Typology: Loeschcke VIII / Bailey Q (x) / Deneauve VIII B
Production Area: Northern Italy / Local
Chronology: 3rd century
Mould-made clay lamp broken in antiquity with a heart-shaped nozzle, a plain discus without an air-hole, pierced handle, and a wide rounded shoulder decorated with two rows of globules between raised edges (Supplementary Fig. S11). The lamp stands on a base ring and does not present any production seal or slip cover[14,15].

Burial 823

Mid-adult female. The body was deposited in a wooden box, as testified by the recovery of at least 14 iron nails, surrounded by a series of pebbles and stones. The body is oriented NE-SW (head NE) (Supplementary Fig. S12) and was deposited with a rich series of objects, which include a clay lamp (Supplementary Fig. S13), five bone pins (Supplementary Fig. S14), one copper-alloy coin, one glass cup, one glass olpe, and one iron knife, all placed at her feet.

Clay lamp imitation of Bailey Q
Typology: Imitation of Baily Q
Production Area: Local
Chronology: 4th-early 5th century
Clay lamp of possibly local production. An imitation inspired by a Loeschcke VIII / Bailey Q (x) but deprived of the typical heart-shaped nozzle (Supplementary Fig. S13). The body is circular with an air-hole on the side, an unpierced ring handle, and shoulders decorated with rays. The lamp stands on a flat base and does not present any production seal or slip cover.

Hairpins: Three hairpins are fragmented, but one has preserved its head, while the other two are intact (Supplementary Fig. S14). The preservation of three of the heads, showing a cylindrical, rounded shape, allows their identification with the Béal AXX.8 / Bianchi H type[16], typical of the 3rd and 4th century for which other 14 exemplars are known in *Albintimilium* from various *domus* and the baths[17].

Despite the partiality of the finds so far exposed, their chronology is in line with the one proposed for the cemetery (late 2nd-late 4th centuries), testifying to the survival of the practice of depositing significant objects with the deceased. There is clearly a preference for objects related to illumination, such as clay lamps and containers (either in ceramic or glass). Jewellery is rarely attested, while there is a survival of the practice of leaving coins to pay for transit in the pagan underworld. Only one of the skeletons with funerary goods was deposited in a simple pit (211). The other individuals were all buried in at least a wooden box or more elaborate forms of depositions. The fact that at least four (211, 435, 509, and 823) out of seven of the adults displaying funerary depositions show female osteological character, while of the three others, two (386 and 445) are also potentially female, denotes some form of either privileged access to this kind of rite or sex-based preferences to the old pagan traditions, usually associated with cremations.

Supplementary Note S2: Osteological Methods

All available individuals from the Necropolis del Sottopasso, currently stored in the depot of the Soprintendenza in Ventimiglia, underwent osteological analysis (n=43) with the permission of the Ministero della Cultura - Soprintendenza Archeologia, Belle Arti e Paesaggio per le Province di Imperia e Savona.

Ten individuals had previously been analysed for a preliminary anthropological report by Capitanio[18]. However, these ten individuals were re-analysed for the purpose of this study, to have comparable data for all individuals. All individuals were analysed using well-established anthropological methods.

Biological sex of adult individuals was assessed using morphological traits in the pelvic girdle[19–21] and skull[22,23], with a preference for pelvic data if available, as the skull will become more masculine with older age in females (> 55 years) and younger males (< 22 years) might not yet have developed secondary sex characteristics in the skull[19,24,25]. Additionally, this sex assessment was corroborated by long bone measurements, especially whenever morphological traits were not well preserved, relying on metric standards developed on a modern Italian assemblage with an accuracy between 80 and 90%[26]. Sex estimation was not attempted for non-adult individuals.

Definite females and probable females were pooled into one female group, while definite males and probable males were pooled into one male group. Individuals with ambiguous features or no estimated sex were excluded from any analyses pertaining to sex-based differences.

Estimates of age-at-death in adults relied on evaluating the morphology of the pubic symphysis[27,28] and auricular surface of the os coxae[29], resorting to dental attrition[30] when necessary. Additionally, Transition Analysis 3 (TA^3)[31] was utilised. This method generates a maximum likelihood point estimate (MLPE) for age-at-death with a 95% confidence interval from the scored skeletal traits. The probability that a skeleton of a certain age will display an observed trait is estimated from a reference sample. Together with a plausible mortality distribution, this probability estimate is integrated into Bayes' Theorem to produce the MLPEs. This method has proven to be more accurate when it comes to estimating the age-at-death of individuals aged 40 years or older[32]but tends to be less accurate for young adults.

For non-adult individuals, age estimation included considerations of dental formation and eruption[33–35]. This was combined with the assessments of epiphyseal fusion[36] and measurements of long bones[37,38]. For pre-term/fetal and neonate individuals, we included measurements of cranial bones[36,39].

Individuals were then assigned to one of nine broader age ranges according to Buikstra and Ubelaker[19] for adults and Scheuer and Black[40] for non-adults (Supplementary Table S2).

Adolescents were included in the adult dataset and sampled for stable isotopes. Although skeletally immature, adolescents occupy a transitional phase between childhood and adulthood, as evidenced by Roman Law, where childhood ended around 12 years for girls (with marriage) and 14 years for boys (with the removal of the bulla)[41].

The palaeopathological analyses for this article focussed on dental pathologies, in particular those which have been used extensively as a proxy for past dietary patterns due to their association with high carbohydrate and protein consumption, were recorded, acknowledging their multifactorial aetiology: carious lesions and calculus. Other pathological alterations of the cranial area and the postcranial skeleton were also recorded. These will be reported in a forthcoming publication.

Supplementary Note S3: Carbon & Nitrogen Isotope Analysis

S3.1 Laboratory Procedures for Carbon & Nitrogen Isotope Analysis

Collagen was extracted from human and animal bones using the established laboratory procedure of the Department of Bioarchaeology at the University of Warsaw, following the method outlined by Longin[42] with some modifications. Bone fragments, ranging from 400 mg to 600 mg, underwent manual abrasion, followed by demineralisation in 0.3 M aqueous HCl at room temperature until the mineral components were dissolved. Subsequently, the samples were washed with deionised water, gelatinised in hydrochloric acid solution (pH 3) at 70°C for 48 hours, filtered using Ezee Filter separators, and then frozen. Finally, the samples were subjected to freeze-drying.

Extracted Collagen was then sent to the Vilnius Radiocarbon Laboratory, where it was analysed using an Elementary Isoprime Vision Mass spectrometer connected to a Vario Isotope Cube elemental analyser.

Measurement of elemental concentration was standardised based on acetanilide. Samples were measured against internal (acetanilide) and international (USGS40 and USGS41a) standard materials. Measurement error, expressed as 1 sigma, was estimated based on the repeated measurements of internal standard material (better than ± 0.2‰ for $\delta^{13}C$ and ± 0.3‰ for $\delta^{15}N$).

For further analysis, samples with carbon concentration greater than 13%, nitrogen concentration greater than 4.8% and atomic C/N ratio between 2.9-3.6 were accepted[43–45].

The reported isotopic values in this context represent the ratio of the heavier isotope to the lighter isotope (e.g., $^{13}C/^{12}C$ or $^{15}N/^{14}N$), expressed as δ values in parts per mille (‰). These δ values are relative to internationally defined standards for carbon (VPDB: Vienna Pee Dee Belemnite) and nitrogen (AIR). The calculation follows the equation [$\delta = (R_{sample} – R_{standard})/R_{standard} \times 1000$][46].

S3.2 Faunal Isotope Values Results and Discussion

Faunal bulk bone collagen $\delta^{13}C_{collagen}$ and $\delta^{15}N_{collagen}$ measurements range from -22‰ to -19.2‰ and 3.5‰ to 10.5‰ respectively (Supplementary Dataset S2). Sampling included various taxa.

Overall, the animal values indicate a C_3 terrestrial diet with only small variation and no evidence of direct C_4 plant consumption of animals.

Chickens are known to feed on insects, which have a high protein content[47,48], and their consumption could, therefore, lead to elevated $\delta^{15}N_{collagen}$ values. Similarly, the consumption of food scraps and their excrements could also explain the high $\delta^{15}N_{collagen}$ values[49]. The wide range of $\delta^{15}N_{collagen}$ values amongst pigs can most likely be attributed to varying animal husbandry strategies and, therefore, different shares of animal protein in their diet, or they may be attributed to import of animals from regions with varying $\delta^{15}N_{collagen}$ values. The lowest $\delta^{15}N_{collagen}$ amongst the pigs suggests a higher share of C_3 plants in their diet, as could be the case for an animal

foraging, while higher $\delta^{15}N_{collagen}$ values suggest a higher share of animal proteins. Particularly for pigs, various husbandry strategies have been highlighted through archaeological and zooarchaeological studies. They can be kept in close proximity to human settlements in enclosures or stables (e.g. found at the villa of Settefinestre[50]), where they would feed more on scraps of human food, potentially leading to elevated $\delta^{15}N_{collagen}$ values, or they can be kept off-site in natural pastures[51,52], where they would feed on a higher share of plants which would keep their $\delta^{15}N_{collagen}$ values lower. Strategies along this spectrum were likely practised in parallel, potentially explaining the variability in values visible in *Albintimilium*[52–54].

One of the two horses was assessed to be juvenile, which could be an additional explanation for the high $\delta^{15}N_{collagen}$ values of this particular individual. Since this individual could have been still suckled, it was excluded from the dataset.

High average $\delta^{15}N_{collagen}$ values of two horses could be associated with feeding on ^{15}N-enriched plants, such as oats[55] or plants from heavily manured fields.

Supplementary Note S4: Bayesian Modelling Background ReSources

ReSources, an upgraded version of the Bayesian Software FRUITS[56], is an R-based application for implementing Bayesian mixing models for isotope-based dietary reconstruction developed within the Pandora and IsoMemo initiatives (https://isomemoapp.com)[57]. ReSources includes descriptions of dietary routing mechanisms, diet-to-consumer isotopic offsets, food macronutrient concentrations, and corrections for isotopic differences between edible macronutrients and measured isotopic values in food remains[56,58,59].

We used ReSources to reconstruct human diets for the individuals from Albintimilium by quantifying caloric and macronutrient contributions of each defined food source.

For dietary modelling, we used two primary models:

The first model, as employed in various recent publications[59–61], relied on broader food categories rooted in chemical classifications, trophic categories or habitat divisions: C_3 plants, C_4 plants, C_3 terrestrial animals, marine resources and freshwater resources.

The proximity of Ventimiglia to the seashore, as well as its location between two river streams (Nervia and Roya), both with available aquatic resources in modern times, necessitated the use of both aquatic categories – marine and freshwater resources.

For the second model, we decided to further split up the C_3 animals into ovicaprids, cattle, pigs and poultry to make full use of available zooarchaeological and historical data. C_3 plants, C_4 plants, freshwater and marine resources were kept the same as in the first model.

This food isotopic references were collected/defined as follows:

Samples from eleven animals (Cattle, Ovicaprids, Pigs, and Poultry) collected from the same excavation as the human individuals provided the food isotopic references for terrestrial fauna. We acknowledge the small sample size of these baseline values,

however, in an attempt to accurately represent locally available isotope values, we decided against combining the local fauna with data from other sites. We did, however, compare the values to other samples from Italy from the CIMA[62,63] and Isotòpia[64,65] databases to ensure their overall accuracy.

We hope that additional sampling in the future will extend this local database and allow for modelling with a more extensive baseline.

For wheat, barley and legumes, we relied on published values from Lattara[66] and Santa Severa[67], representing distinct chronological periods and geographical regions, which are, however, subject to similar meteorological phenomena (e.g. similar mean annual precipitation) as *Albintimilium*. These exhibited overlapping isotopic values with no significant differences in Carbon or Nitrogen, leading to them being pooled. We opted to exclude remains from Portus Romae[68] as these grains represent products from one of the primary ports of the Roman Empire, likely sourced from various regions. Relying on the remains from Portus Romae would inadequately capture the locally available C_3 plant signature due to the substantial variability in wheat values, likely arising from diverse wheat origins that were imported to the capital[68]. Several historical sources and available palynological studies have shown that grains were primarily produced locally[9,69–71].

Due to the lack of contemporary isotopic samples or values for C_4 plants, we relied on isotopic measurements from Bronze Age Greece[72]. This approach mirrors the methodology employed by Cocozza and colleagues[63,73].

For freshwater and marine resources, given the absence of locally available remains in the Ventimiglia area, we amalgamated data from the CIMA[62,63] and Isotòpia[64,65] databases from different areas of the Roman Empire (Italy, Switzerland, France) with more recently published results from Southern France[74]. A combination of fish values from different places in the Mediterranean is further supported by the fact that fermented fish sauces (liquamen or garum) were widely used in Roman cuisine. These were not necessarily produced locally but often imported. Until the late 4th century, the primary source for fish sauces (amongst other things) was Spain, after which the majority of imported produce came from North Africa, as evidenced by the analysis of transport vessels in the region[75–80].

We acknowledge that the mean values of freshwater resources we obtained through combining these sources slightly differ from previous studies and show large standard deviations. This is, however, due to the inclusion of a new large dataset on (early) medieval fish remains from the area around Marseille[74], the geographically closest available dataset for this study. This dataset includes a significant amount of eel, a catadromous species, which shows a large variety of isotope values. We deemed the inclusion of this dataset important because eel did represent part of the consumed freshwater resources, as evidenced by several Graeco-Roman writings on dietary health, the recovery of their remains in archaeological sites and the fact that eel is one of the species that are currently found in the waters around modern-day Ventimiglia.

$\delta^{13}C_{collagen}$ and $\delta^{15}N_{collagen}$ values for food sources are listed in Supplementary Table S3 for the 5-group and 8-group models with a plot (Supplementary Fig. S16a-b) showing their distribution, respectively.

In order to obtain $\delta^{13}C$ and $\delta^{15}N$ values for macronutrient components in food (such as carbohydrates/lipids versus protein), we implemented offset corrections comparing the measured material (e.g., bone collagen) with the edible nutritional component of the corresponding food group (e.g., muscle meat protein). We applied the offset corrections given in Supplementary Table S4 with uncertainties for macronutrient isotopic values following Fernandes and colleagues[59] (corrected in Soncin and colleagues[81]) to macronutrient isotopic values.

This resulted in the isotopic reference values used in the Bayesian modelling presented in Supplementary Table S5.

Additionally, we employed macronutrient caloric concentration values as reported in Fernandes and colleagues[58] and Cocozza and colleagues[63] and used doubled uncertainty values as reported in Cocozza and colleagues[63]:

For C_4 plants, the concentrations were: protein: 10±5%; carbohydrates/lipids 90±5%. Due to the inclusion of legumes and pulses into the C_3 plant group and their higher protein content, we decided to increase the protein content of this group, as reported in Cocozza and colleagues[73], to: 15±10%, carbohydrates/lipids 85±10%. For animal produce, the concentrations were as follows: terrestrial animals: protein: 30±5%; carbs/lipids 70±5%, aquatic animals: protein: 65±10%; carbs/lipids 35±10%.

Within the Bayesian model, we also accounted for dietary routing mechanisms. Based on previous studies[56,59], we assumed that bone collagen $\delta^{15}N$ derived 100% from dietary protein, while we considered that the isotopic signal of bone collagen $\delta^{13}C$ was routed: 74±4% derived from dietary protein and 26±4% from carbohydrates/lipids.

Additionally, we included isotopic offsets between diet and human tissues, which we defined as 5.5±0.5‰ for $\delta^{15}N$ bone collagen[59] and 4.8±0.5‰ for $\delta^{13}C$ bone collagen[56].

Lastly, to improve the resolution of the dietary estimates, we introduced prior constraints based on non-isotopic dietary evidence relative to (Late) Roman Liguria.

The prior selection process proved to be an assiduous task given the scarcity of non-isotopic dietary evidence for Late Roman *Albintimilium*. However, direct evidence exists regarding the diet of neighbouring cities during this period through palynological, zooarchaeological studies and historical writings, and we thereby selected the priors based on this currently available information. This approach was made under the assumption that similarities in diet are to be expected in the same region. An important remark that should be noted is that the model may yield somewhat inaccurate results induced by the possible biases in the priors. Nonetheless, the Bayesian framework allows correcting such biases with evidential updates as data are introduced. Any potential inaccuracies are, therefore, being corrected with the gathered isotope data. Incorporating more data, which is expected to become increasingly available in the future, should further improve the dietary distribution given by the posterior for any future analyses.

For both models, the following priors were utilised:

Based on studies on agricultural yield[82] and historical writings about the area from various periods[83], we assumed that the majority of calories was provided by C_3 and C_4 plants, over animal products: **(C_3 Plants + C_4 Plants) > (C_3 animals + Marine**

Resources + Freshwater Resources) / (C_3 Plants + C_4 Plants) > (Ovicaprids + Cattle + Pig + Poultry + Marine Resources + Freshwater Resources).

In turn, due to the higher protein availability in animal produce, we assumed that more protein derived from animal produce than from plants: **(C_3_Protein + C_4_Protein) > (C_3animals_Protein + MarineResources_Protein + Freshwater Resources_Protein) / (C_3_Protein + C_4_Protein) > (Ovicaprids_Protein + Cattle_Protein + Pig_Protein + Poultry_Protein + MarineResources_Protein + Freshwater Resources_Protein)**

Based on historical sources (e.g., Strabo or Diodorus of Sicily as referenced in the main text) about the wider Ligurian region and archaeobotanical and palynological studies from several different archaeological contexts in *Albintimilium*[9,69,70] and other (coastal) Ligurian sites[71,76,84] we assumed that C_3 plants were consumed in larger amounts than C_4 plants. In the assemblage from Cavo Hill, charred macro remains of C_3 plants make up more than 70% of the recovered and analysed seeds, while C_4 plants (*Panicum miliaceum* and *Setaria Italica*) make up only about 7%[69]. This pattern is also observable in agricultural writings from the late 19th century dealing with the area around Ventimiglia, where more importance is placed on C_3 plants such as wheat bread, chickpea flour bread, chestnuts, lentils, beans and other legumes[83]. Similar patterns were observed by Ruas[85], Favia and colleagues[86] and Cocozza and colleagues[73]: **C_3 Plants > C_4 Plants**

Due to the location of the site at the seashore and in between the Rivers Nervia and Roya, a higher availability of fresh fish and molluscs for individuals living in *Albintimilium*, compared to inland settlements, is plausible, although this is not mentioned in any available (contemporary) primary sources. Zooarchaeological studies from *Albintimilium*[87] and other regional sites[88], include molluscs and very limited amounts of fish remains. However, it is conceivable that due to the seasonality of fish and a decrease in fish yield during colder winter months, fish produce was consumed less than C_3 animals. Dairy and meat of C_3 animals could have been available all year round, while fish and molluscs might have been scarcer or more difficult to procure during the winter months. Agricultural writings from the late 19th century focusing on workers in Liguria on the coast put very little emphasis on fish consumption: if at all, fish was only consumed occasionally in the form of dried stockfish[83]. **C_3 Animals > Freshwater Resources** and **C_3 Animals > Marine Resources / (Ovicaprids + Cattle + Pig + Poultry) > Freshwater Resources** and **(Ovicaprids + Cattle + Pig + Poultry) > Marine Resources**

Additionally, for the 8-source model, we relied on the following priors:

Available zooarchaeological studies from *Albintimilium*[87] and other sites in Northern Italy and Southern Gaul[89,90] provided us with several insights into livestock management in the area at the time.

A study by Corbino and colleagues[91] found higher rates of Pig than Poultry in all investigated Late Roman sites in Italy. This is also confirmed *Albintimilium,* with available zooarchaeological studies reporting very low numbers of Poultry remains[87,92]. We therefore added a prior: **Pig > Poultry**

Zooarchaeological studies in, e.g. Filattiera-Sorano[93,94], Corti[89], Noli[90], Arles and Olbia[88] have found proportionally low numbers of horse and cattle remains. Although

the number of cattle remains is higher in some contexts from *Albintimilium*[87] directly, they are in most cases still less than those of pigs[87,92]. Additionally, the slaughter age of cattle and pathologies associated with high loads of biomechanical stress still evidence their primary use in agricultural activities. They were mostly only slaughtered at the end of their working cycle[87]. Similar assumptions were made for the remains recovered from the sites[88] mentioned above: **Pig > Cattle**

The full models are available as Supplementary files to this article.

In addition to the plot provided in the main text showing the results of the Bayesian dietary estimates of caloric contribution, Supplementary Figure S17a-f also shows the results for female individuals from Phase VII (Supplementary Fig. S17b and e) and male individuals from Phase VII (Supplementary Fig. S17c and f). It is observable that the estimates for aquatic resources (both freshwater and marine), as well as estimates for resources coming from a C_4 environment, are higher in male individuals than in female individuals. This matches the observations we made in the scatterplot (Fig. 3 in the main text), where male individuals have, on average, both higher $\delta^{13}C_{collagen}$ and $\delta^{15}N_{collagen}$ values compared to the female population.

Supplementary Note S5: Detailed ^{14}C Results and Bayesian Modelling

Two samples, one representing each phase (Individuals 455 and 125), were selected for ^{14}C dating.

Supplementary Figure S22 shows the calibrated ^{14}C dates of the two samples. Although a potential chronological difference in the tails of the dates is observable, the calibrated dates also show the possibility of the individuals being contemporaneous, which opposes the archaeological interpretations of two distinct phases of the necropolis.

In order to reduce uncertainty and narrow the calibrated ranges, we introduced supporting information that carries (relative) chronological information deriving from the archaeological matrix that can be modelled by a Bayesian approach. The applicability of such an approach has been shown, e.g. by Massy and colleagues[95] and Gnecchi-Ruscone and colleagues[96].

We used the software OxCal[97], incorporating two sets of information: 1) the ^{14}C dates of the individuals and 2) the relative position within the archaeological matrix.

The modelling (Supplementary Figs. S23-S24) confirms the likelihood of two different chronological phases, as indicated by the archaeological evidence. Although the 95% dating ranges stayed very similar for both individuals, the probabilities changed slightly with the introduction of additional relative chronological information.

We see a significantly decreased probability of an early third-century dating for individual 125 and an increase in probability for a dating between the mid-4th and early 5th century. Additionally, we see a slightly narrower range for individual 455, reducing the probability of a date extending into the 3rd third of the 4th century CE, but rather dating between the mid-3rd and mid-4th century.

Supplementary Figures

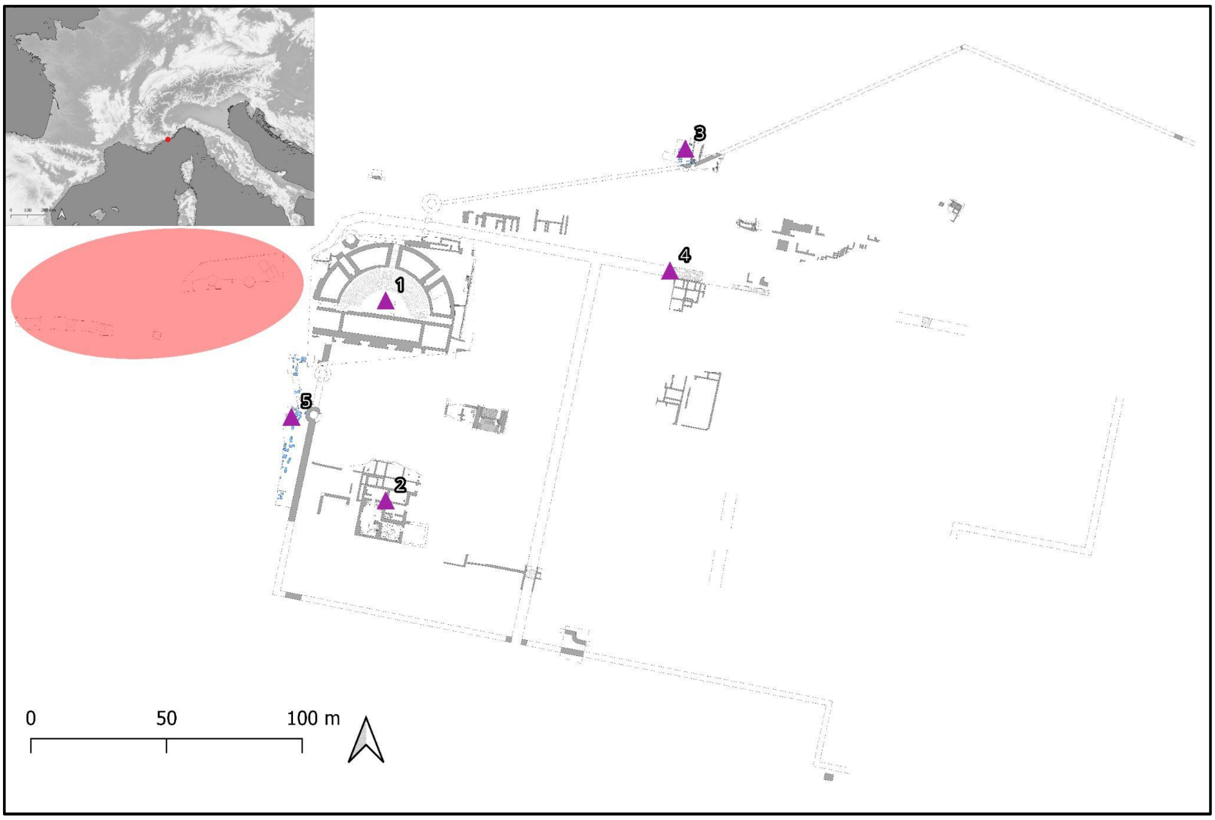

Supplementary Figure S1: A plan of the excavated areas of Albintimilium *showing the Western necropolis (in red), and the location where other funerary areas have been found: 1) theatre; 2) the Roman baths (one infant burial); 3) northern necropolis; 4) decumanus; 5) "Necropoli del Sottopasso". Source: Soprintendenza Archeologia, Belle Arti e Paesaggio per le province di Imperia e Savona with modifications of Alessandro Carabia.*

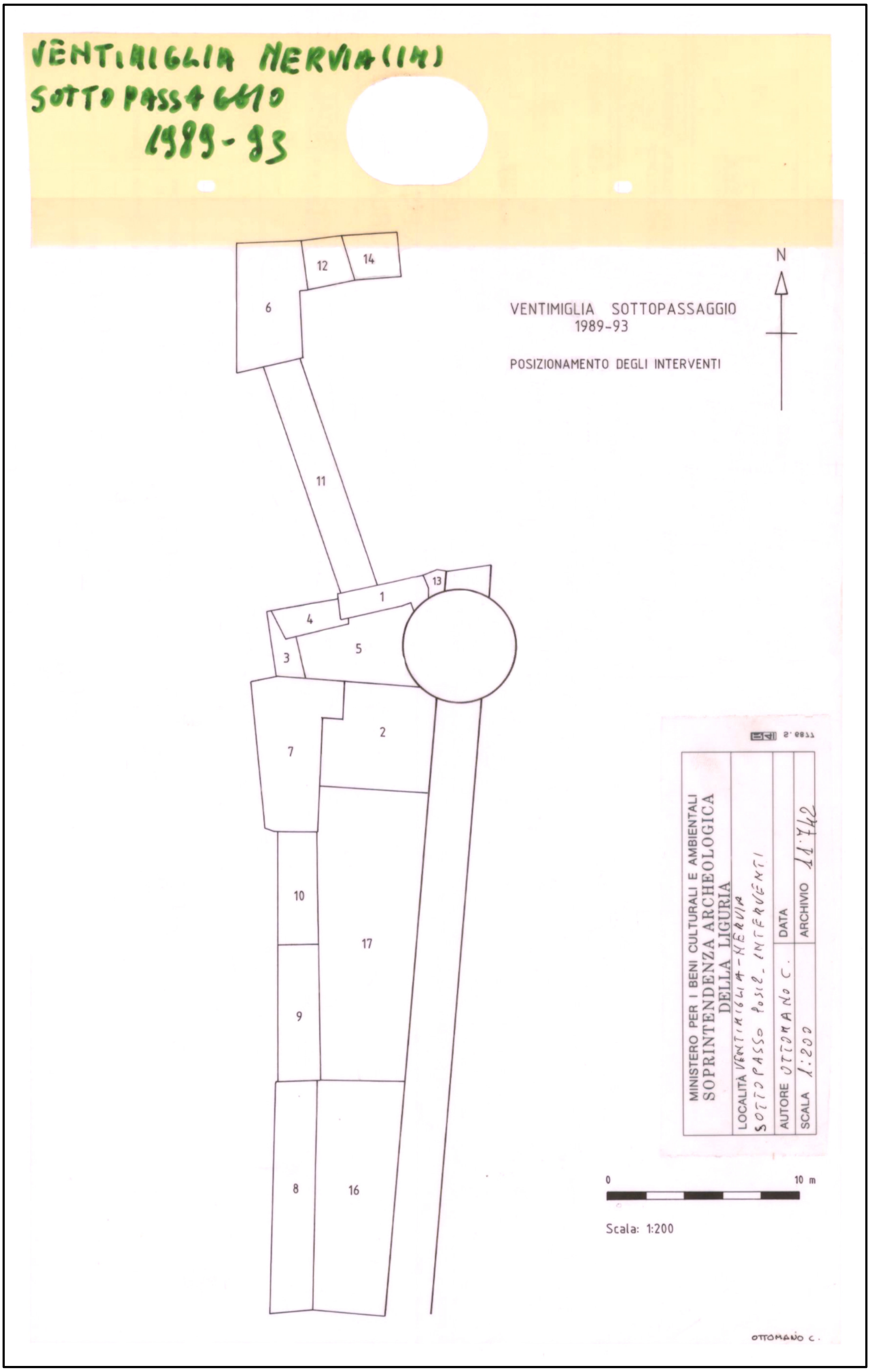

Supplementary Figure S2: *Plan of the excavation area evidencing the distribution and numbering of the "interventi" (trenches). Source: Soprintendenza Archeologia, Belle Arti e Paesaggio per le Province di Imperia e Savona*.

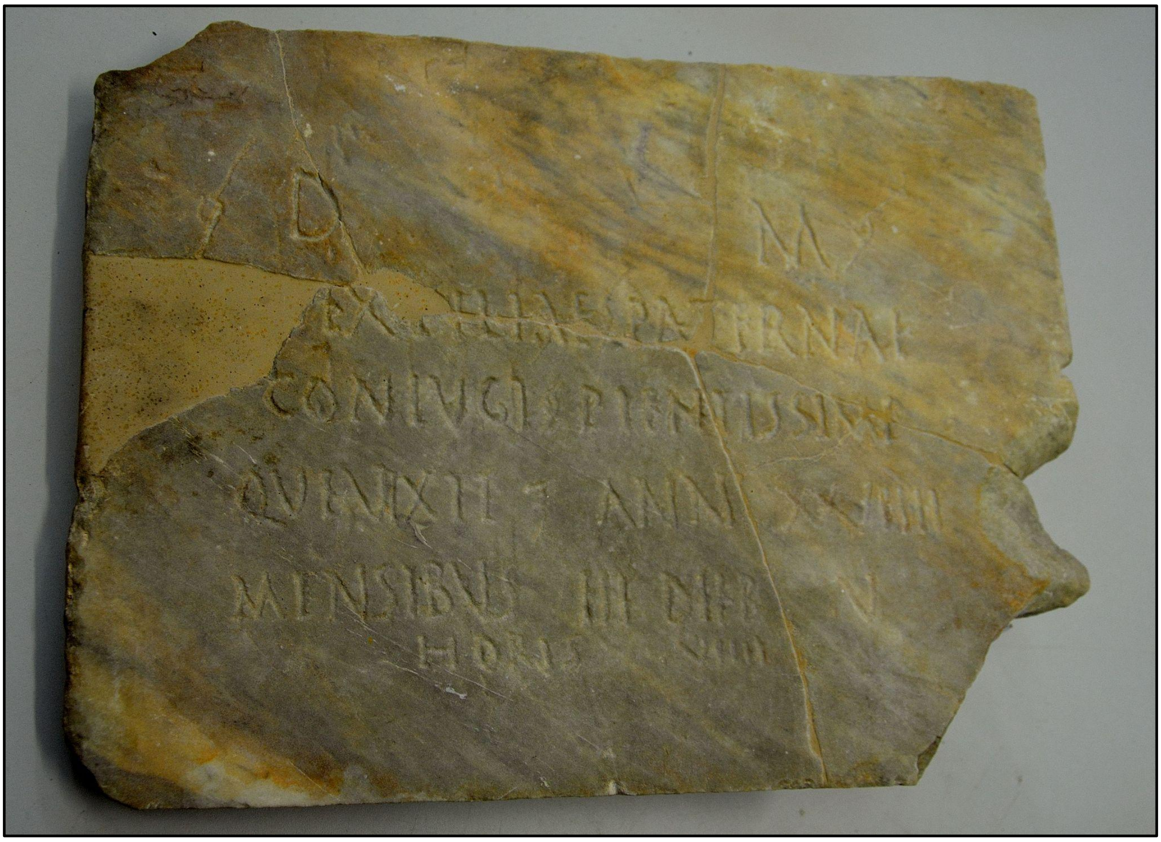

Supplementary Figure S3*: Epitaph of Sextilia Peterna.*
D(is) M(anibus) / [S]extiliae Paternae / coniugi pientissime, / que vixit ann(is) XXVIIII / mensibus III, dieb(us) V, / horis VIIII.
To the Manes. To Sestila Peterna, devotest spouse, who lived twenty-nine years, three months, five days, and nine hours. 29x38.5x2.2 cm – marble
Source: Soprintendenza Archeologia, Belle Arti e Paesaggio per le province di Imperia e Savona. Photo credit: Alessandro Carabia

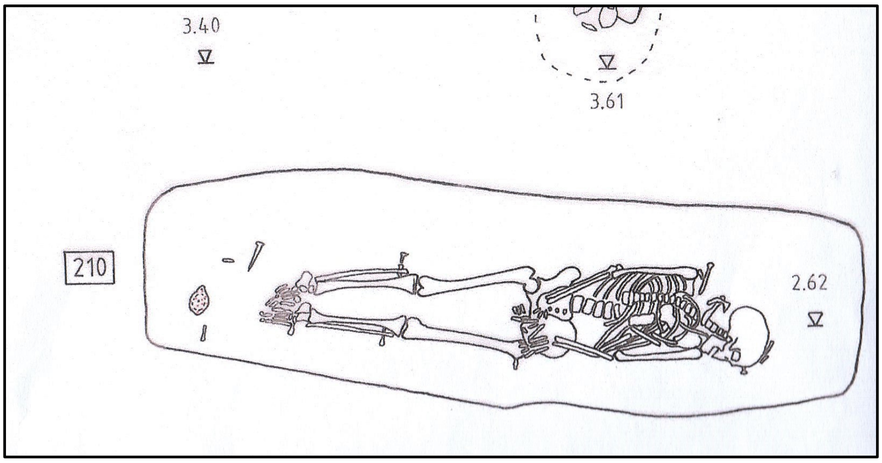

Supplementary Figure S4*: Drawing of burial 210 with details of the skeleton and finds (nails and clay lamp). Source: Soprintendenza Archeologia, Belle Arti e Paesaggio per le province di Imperia e Savona*

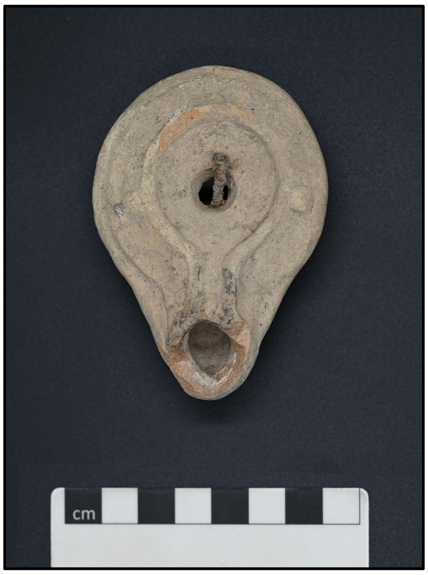

Supplementary Figure S5: *Imitation of Firmalampen X-c deposited in burial 210 at the feet of the skeleton. Source: Soprintendenza Archeologia, Belle Arti e Paesaggio per le province di Imperia e Savona. Photo credit: Alessandro Carabia*

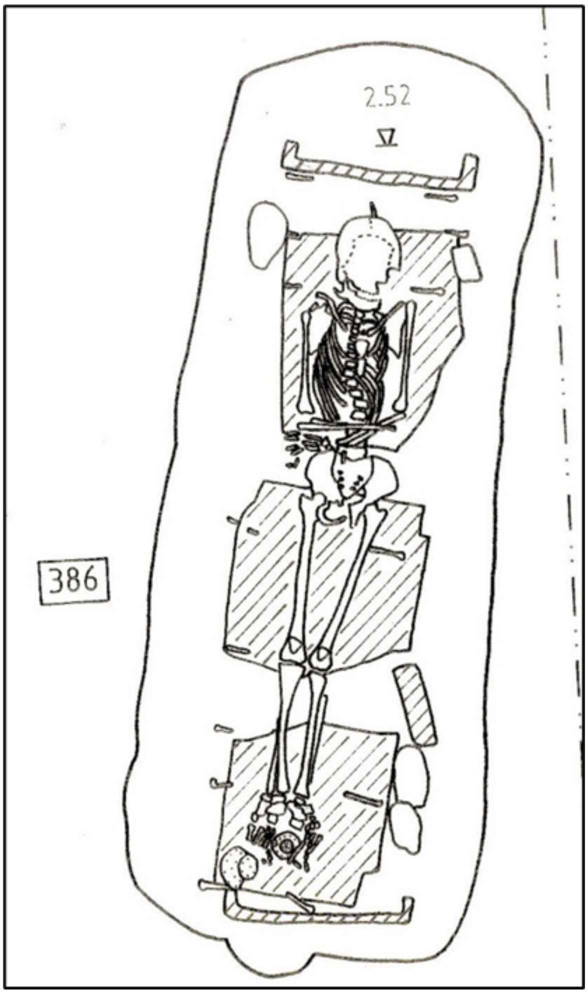

Supplementary Figure S6: *Drawing of burial 386 with details of the skeleton and finds (nails, clay lamp, tiles, and the olla). Source: Soprintendenza Archeologia, Belle Arti e Paesaggio per le province di Imperia e Savona*

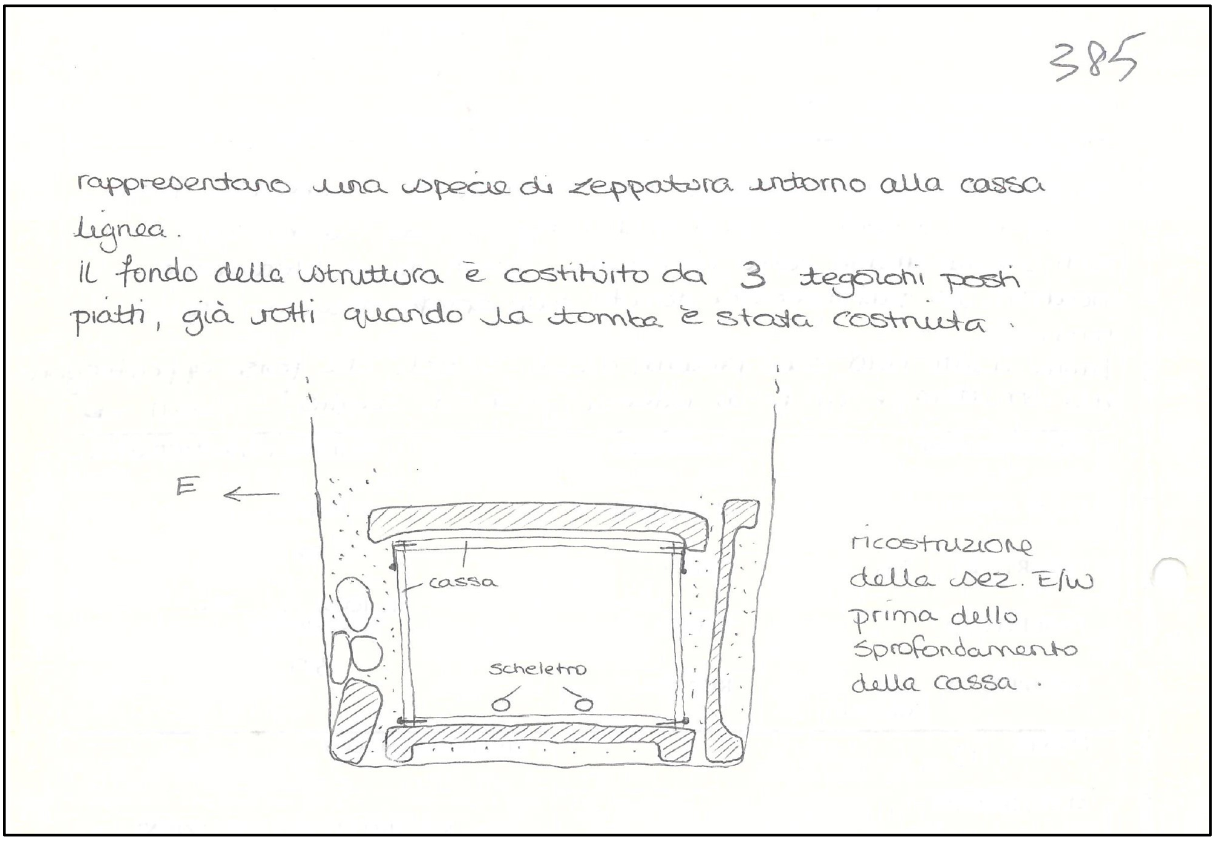

Supplementary Figure S7: *Sketch of a possible reconstruction of the section of the burial from the back of the original context sheet. Source: Soprintendenza Archeologia, Belle Arti e Paesaggio per la province di Imperia e Savona.*

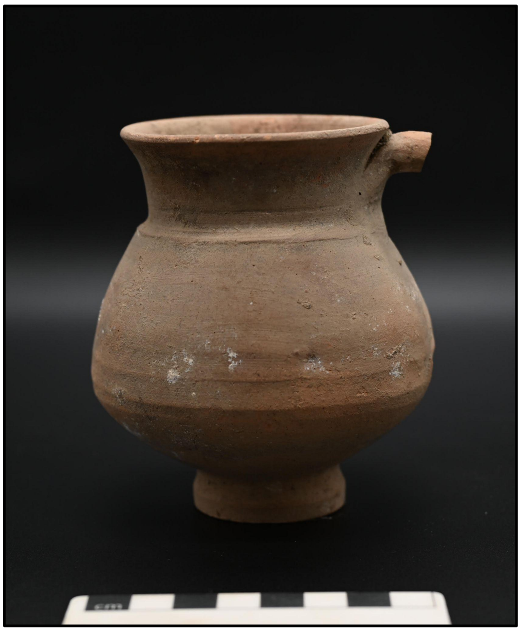

Supplementary Figure S8: *Olla with broken handle found at the feet of deposition 386. Source: Alessandro Carabia Source: Soprintendenza Archeologia, Belle Arti e Paesaggio per le province di Imperia e Savona. Photo credit: Alessandro Carabia*

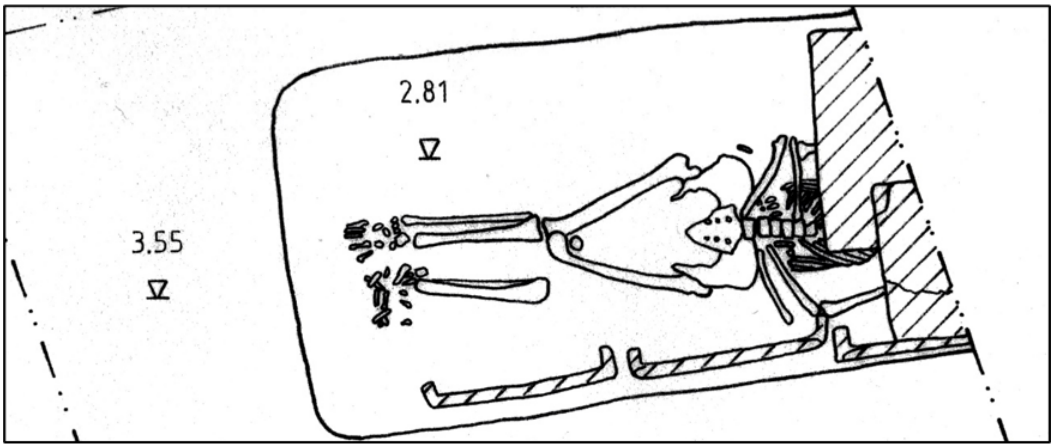

Supplementary Figure S9*: Drawing of burial 435 with details of the skeleton and the tile box. Source: Soprintendenza Archeologia, Belle Arti e Paesaggio per le province di Imperia e Savona*

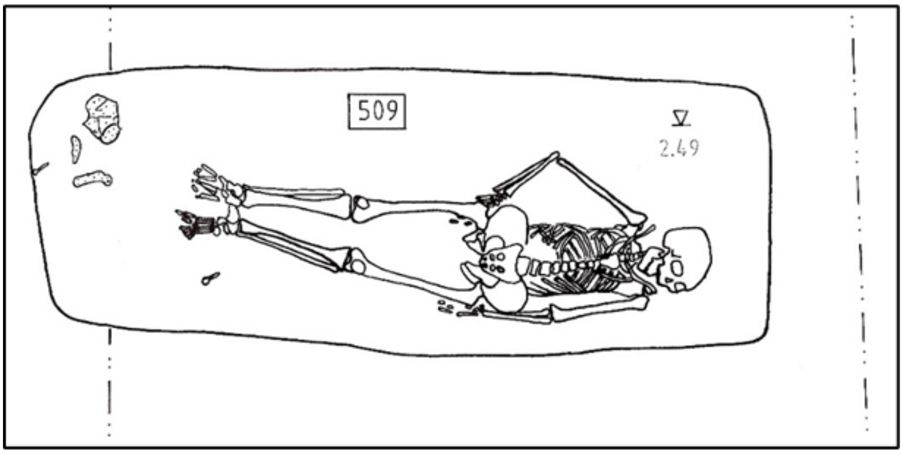

Supplementary Figure S10: *Drawing of burial 509 with details of the skeleton and finds. Source: Soprintendenza Archeologia, Belle Arti e Paesaggio per le province di Imperia e Savona*

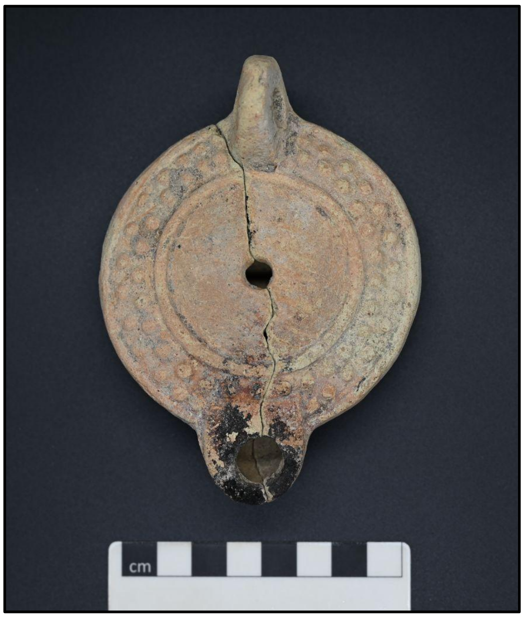

Supplementary Figure S11: *Clay lamp Deneauve VIII B/Baily Q (x) broken in antiquity and deposited with the skeleton from burial 509. Source: Soprintendenza Archeologia, Belle Arti e Paesaggio per le province di Imperia e Savona. Photo credit: Alessandro Carabia*

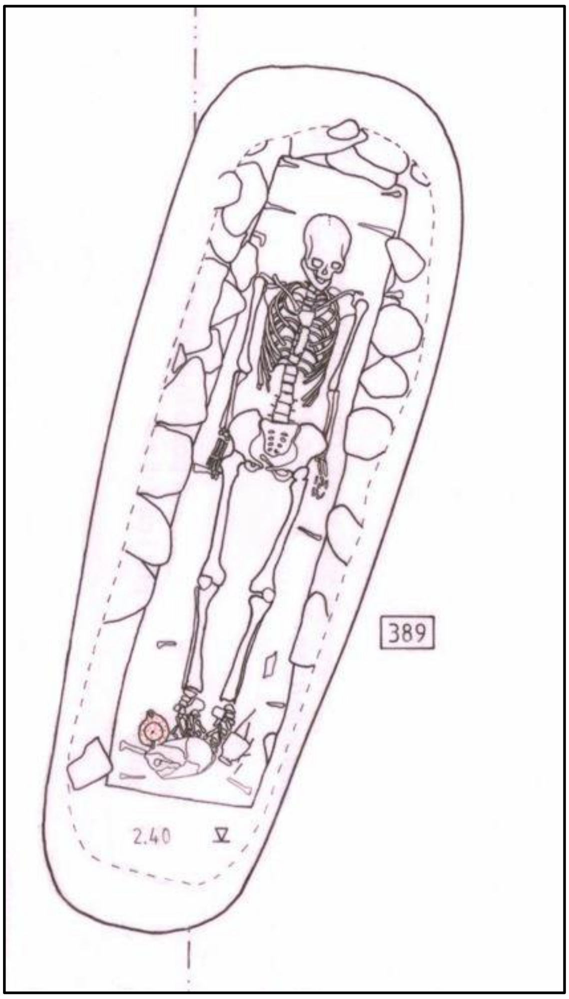

Supplementary Figure S12: *Drawing of burial 823 with details of the skeleton and finds. Source: Soprintendenza Archeologia, Belle Arti e Paesaggio per le province di Imperia e Savona*

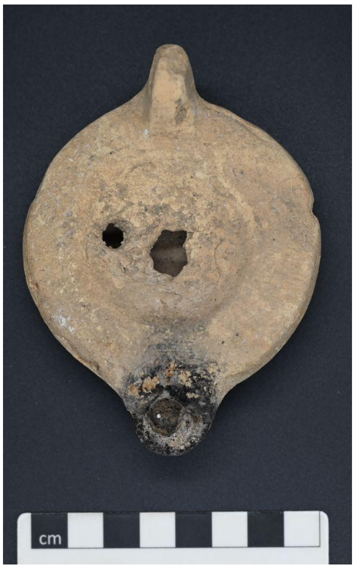

Supplementary Figure S13: *Clay lamp, imitation of a Bailey Q deposited in burial 823. Source: Soprintendenza Archeologia, Belle Arti e Paesaggio per le province di Imperia e Savona. Photo credit: Alessandro Carabia*

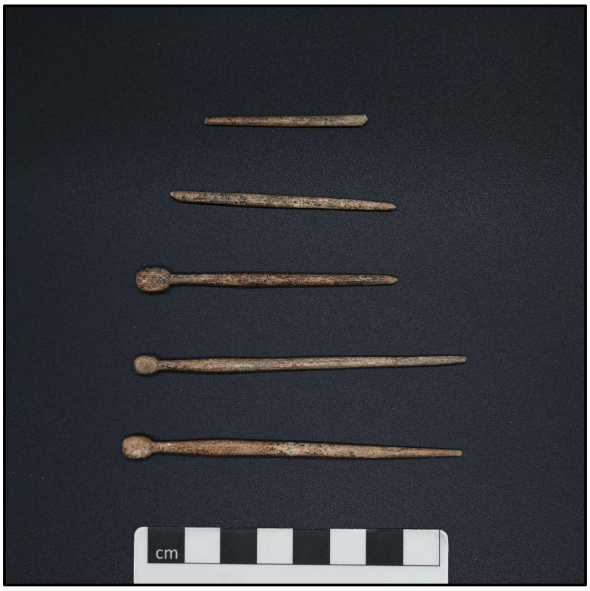

Supplementary Figure S14: *Set of hairpins from burials 823. Source: Soprintendenza Archeologia, Belle Arti e Paesaggio per le province di Imperia e Savona. Photo credit: Alessandro Carabia*

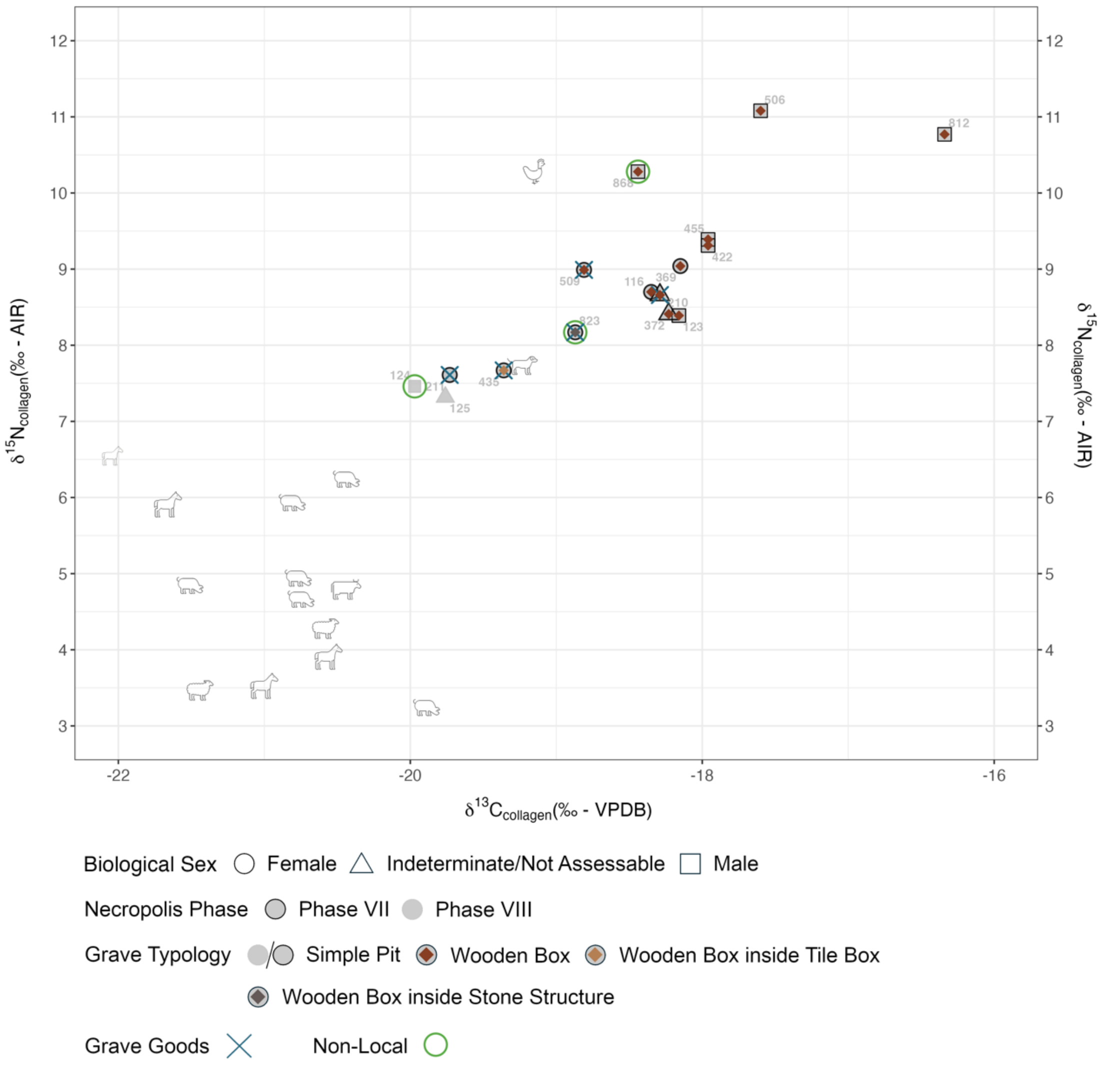

Supplementary Figure S15: *Scatterplot showing* $\delta^{13}C_{collagen}$ *and* $\delta^{15}N_{collagen}$ *values of humans and animals from* Albintimilium*. Humans are divided into their respective phases and biological sex. Additionally, the plot includes information on grave typologies, the presence of grave goods and whether an individual is considered non-local based on their* $^{87}Sr/^{86}Sr$ *values.*
Animals are presented by icons. A juvenile horse sample, excluded from further analysis, is shown with a smaller, lighter icon.

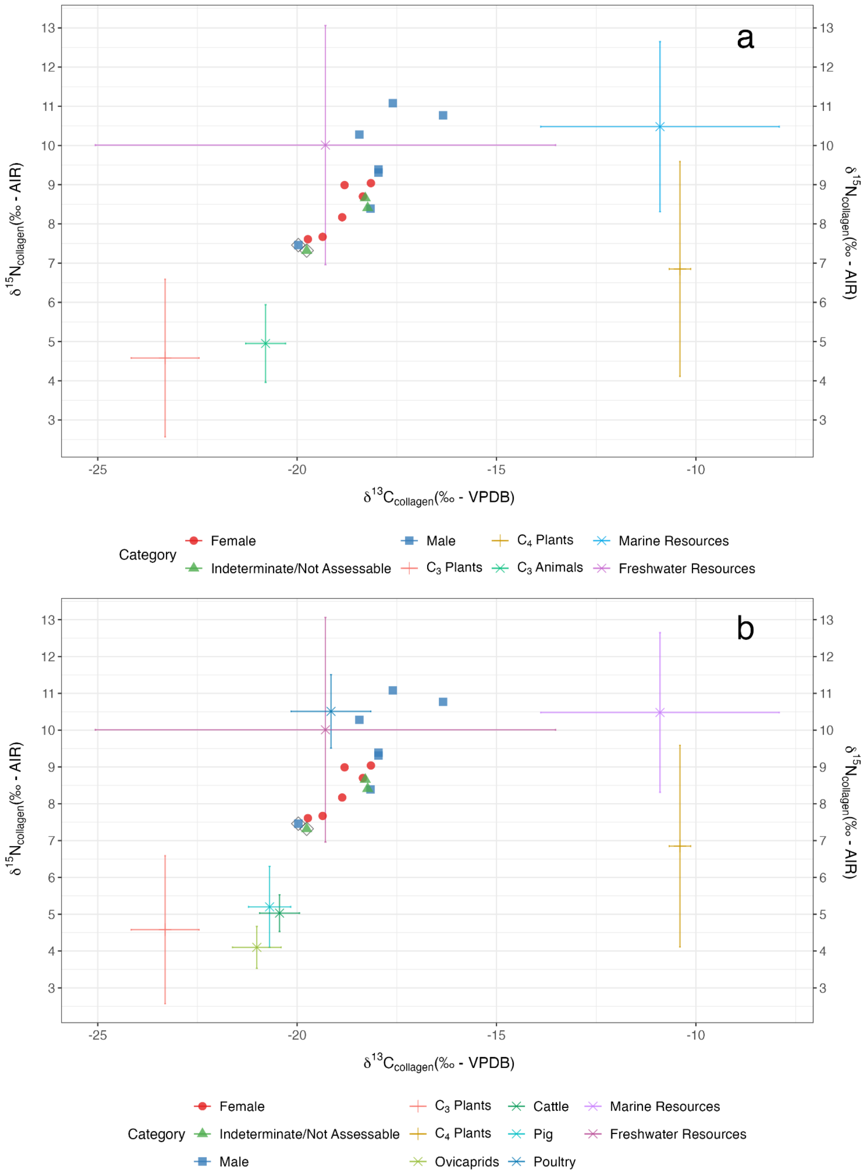

Supplementary Figure S16a-b. *Scatterplot showing individuals from* Albintimilium *(filled circles, triangles and squares) together with the mean and standard deviation of potential food sources used in the ReSources model: (a) 5 food groups, (b) 8 food groups. Individuals from Phase VIII are highlighted with a grey diamond around them.*

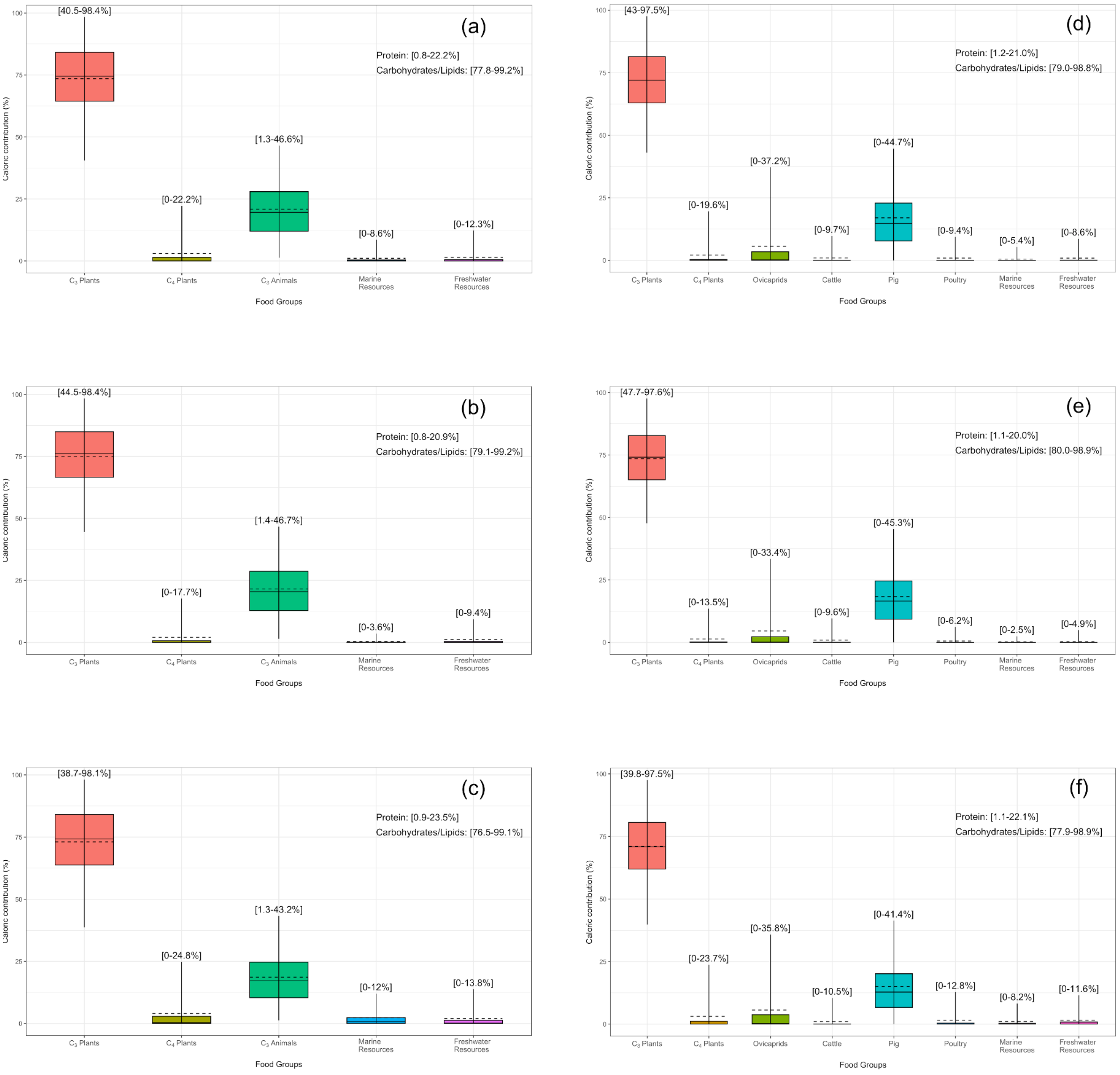

Supplementary Figure S17a-f: *Bayesian estimates of dietary caloric contribution. a) - c) 5 food groups (C_3 plants, C_4 plants, C_3 animals. Marine, Freshwater) - All individuals (a), Female individuals Phase VII (b), Male individuals Phase VII (c); d) - f) 8 food groups (C_3 plants, C_4, plants, Ovicaprids, Pig, Cattle, Poultry, Marine resources, Freshwater resources) - All individuals (d), Female individuals Phase VII (e), Male individuals Phase VII (f).*
Boxes and whiskers represent 68% and 95% credibility ranges, respectively. Horizontal continuous lines represent the mean and dashed horizontal lines the median. Numbers in brackets represent 95% credible ranges. Also included within the graph are numeric estimates of the caloric contributions of protein versus. carbohydrates/lipids macronutrients.

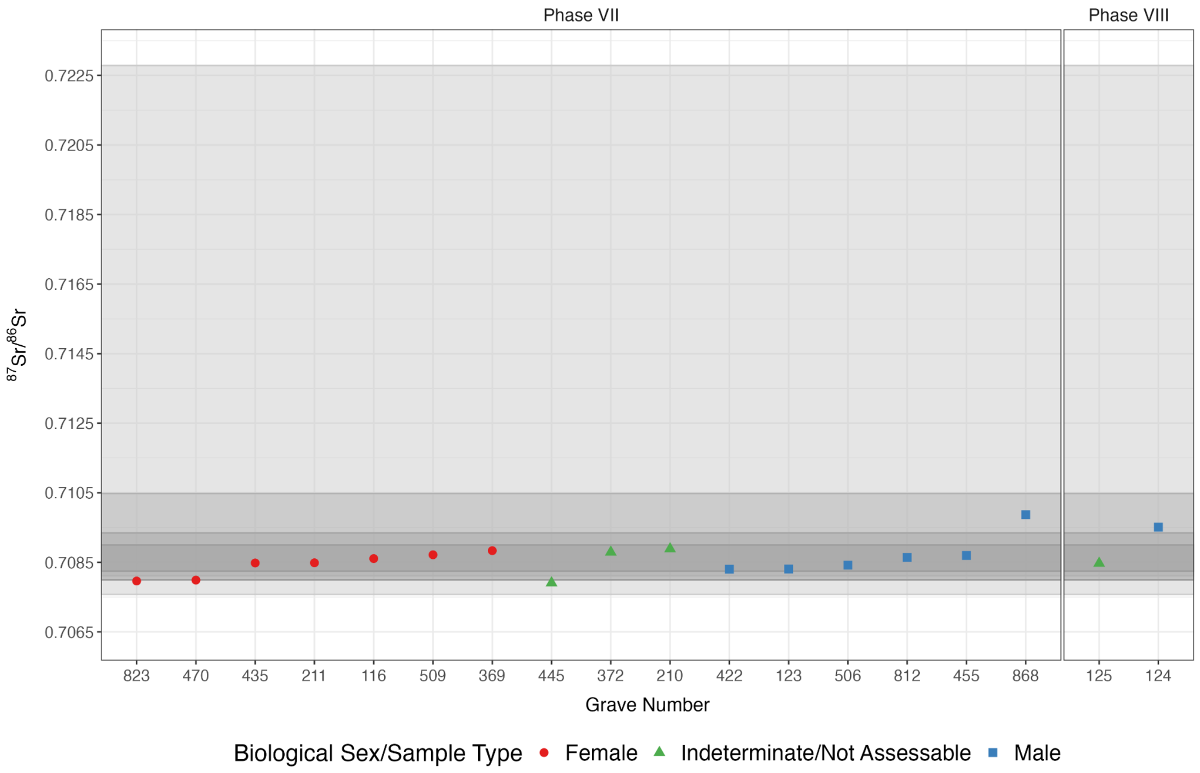

Supplementary Figure S18: *^{87}Sr/^{86}Sr values of humans from* Albintimilium *split into the two distinct burial phases combined with the bioavailable ^{87}Sr/^{86}Sr from the region. The darkest band represents the area directly around the site, the second darkest band represents the environment including samples from Mortola Inferior and Dolceacqua, the second lightest band represents the wider environment along the Ligurian coast, while the lightest band represent the samples collected in Provence and along the French coast.*

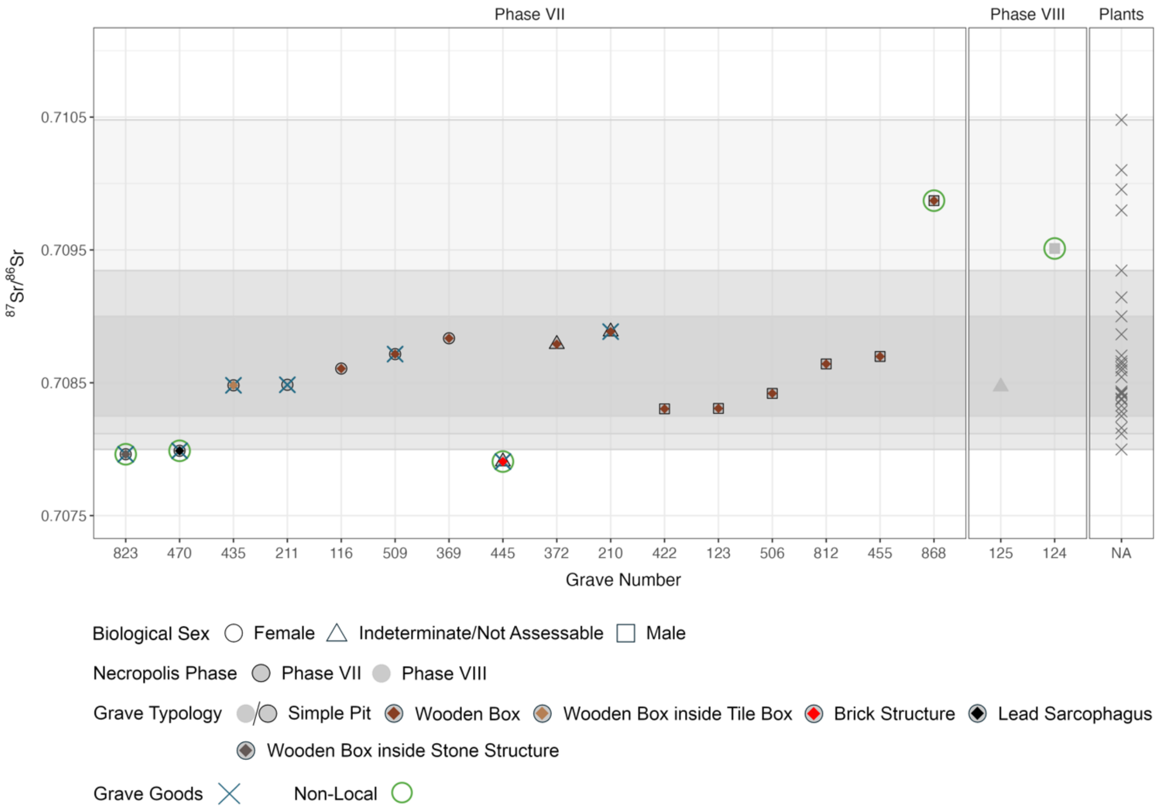

Supplementary Figure S19: *$^{87}Sr/^{86}Sr$ values of humans from* Albintimilium *split into the two distinct burial phases combined with the bioavailable $^{87}Sr/^{86}Sr$ from the Ligurian region. Additionally, the plot includes information on grave typologies, the presence of grave goods and whether an individual is considered non-local based on their $^{87}Sr/^{86}Sr$ values.*

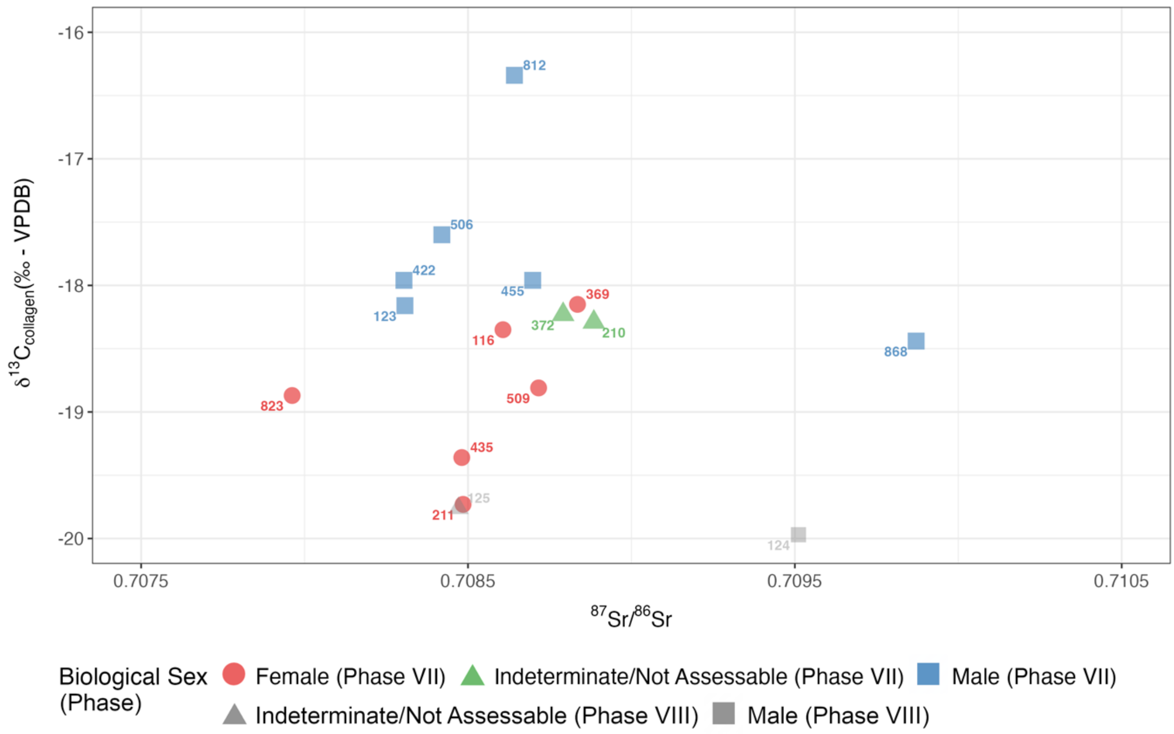

Supplementary Figure S20: *Scatterplot showing $\delta^{13}C_{collagen}$ and $^{87}Sr/^{86}Sr$ values of individuals from* Albintimilium*.*

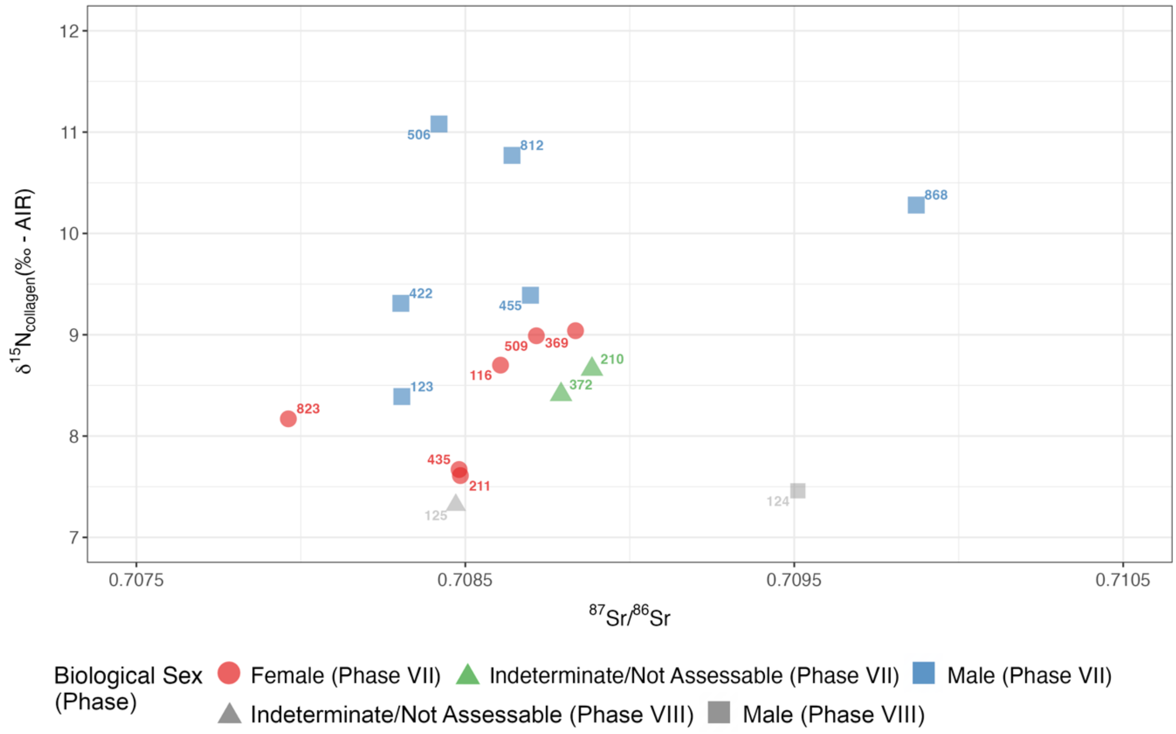

Supplementary Figure S21: *Scatterplot showing $\delta^{15}N_{collagen}$ and $^{87}Sr/^{86}Sr$ values of individuals from* Albintimilium*.*

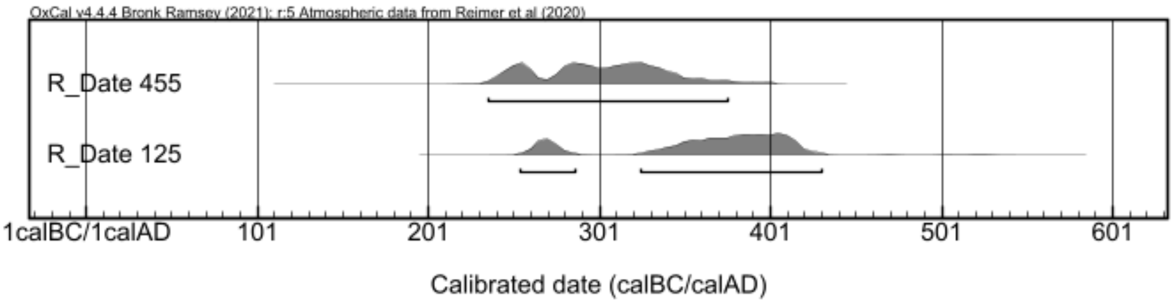

Supplementary Figure S22: *Calibrated* ^{14}C *dates for individuals from* Albintimilium*.*

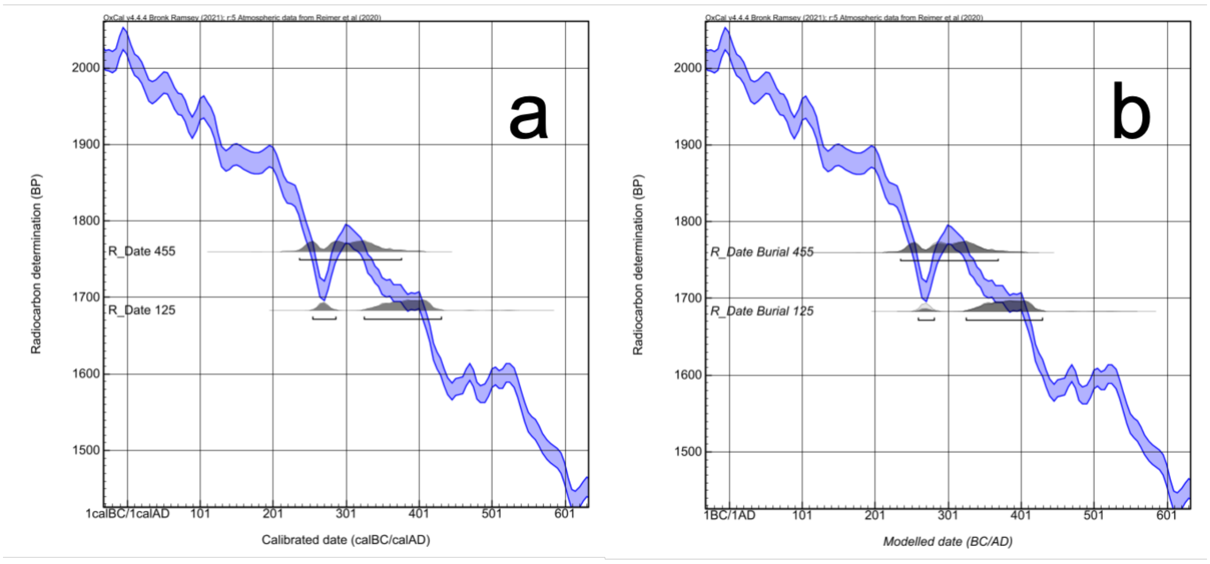

Supplementary Figure S23a-b: *Calibration curves with calibrated (a) and modelled (b) radiocarbon dates from the "Necropoli del Sottopasso" with probability distributions. The area under the curve indicates the probability of dating the individuals to the corresponding time-period.*

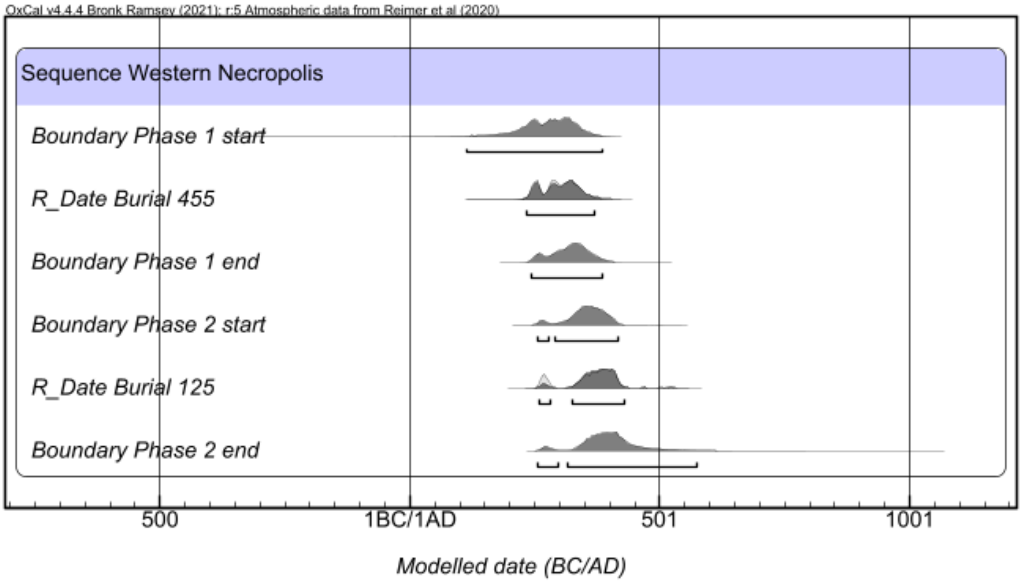

Supplementary Figure S24. *A simplified modelled sequence utilising the two radiocarbon dates and the information on relative chronology derived from the excavation Matrix attributing the two individuals to two distinct phases.*

Supplementary Tables

Supplementary Table S1. *Burials with grave goods. Individuals marked with * were not available for analysis - the individual was categorised as "non-adult" based on the excavation report.*

**Burial Number**	**Burial Number - Ventimiglia sequence**	**Burial Typology**	**Sex**	**Age**	**Grave Goods**
43	231	Enchytrismò s	NA	Non-Adult*	1 necklace bead
68	234	Enchytrismò s	NA	Older Child	1 handled cup in "terra sigillata lucente"
210	246	Wooden box	I	Old Adult	1 clay lamp, 1 iron curved object
211	247	Simple pit	F	Young Adult	1 copper-alloy coin
386	262	Wooden box inside tile box	NA	Adolescent	1 clay lamp, 1 olla
435	266	Wooden box inside tile box	F	Young Adult	1 fragmented clay lamp, 1 necklace bead (glass)
445	267	Bricks structure	NA	Adolescent	2 copper-alloy earrings
509	271	Wooden box	F	Old Adult	1 clay lamp (broken in antiquity), 1 olla (broken in antiquity)
823	274	Wooden box inside stone circle	F	Middle Adult	1 clay lamp, 1 iron knife, 5 bone pins, 1 glass olpe, 1 glass cup, 1 copper-alloy coin

Supplementary Table S2: *Age-at-death categories*

**Non-Adults**[36]		**Adults**[19]	
**Category**	**Age Range**	**Category**	**Age Range**
Pre-Term	< 37 weeks	Adolescent	13 - 17 years
Neonate	Birth - 28 days (38 - 44 weeks)	Young Adult	18 - 34 years
Infant	1 month - 1 year	Middle Adult	35 - 49 years
Young Child	1 - 5 years	Old Adult	> 45 years
Older Child	6 - 12 years		
Non-Adult	<12 years	Adult	18+ years

Supplementary Table S3: *Isotopic values of food sources used in Bayesian dietary modelling. These values do not yet include correction for offsets between edible tissues and food remains (e.g. meat protein or lipids vs bone collagen). Corrected values are given in Supplementary Table S5.* *only used in the 8-food-group model, ** only used in the 5-food-group model.

		**$\delta^{13}C_{collagen}$**	**sd**	**$\delta^{15}N_{collagen}$**	**sd**
C_3 plants		-23.31	0.85	4.58	2.01
C_4 plants		-10.4	0.27	6.85	2.74
C_3 animals**		-20.74	0.5	4.95	0.99
	Ovicaprids*	-21.01	0.61	4.1	0.57
	Cattle*	-20.44	0.50	5.03	0.5
	Pig*	-20.69	0.53	5.2	1.1
	Poultry*	-19.15	1	10.51	1
Marine		-10.9	2.99	10.48	2.17
Freshwater		-19.29	5.77	10.01	3.05

Supplementary Table S4: *Offset corrections for macronutrient isotopic values rounded up to multiples of 0.5*

	**$\Delta^{13}C$**		**$\Delta^{15}N$**
	**Protein-Collagen***	**Energy**-Collagen***	**Protein-Collagen***
Plants	-2	0.5	0
Terrestrial Animals	-2	-8	0
Aquatic Animals	-1	-7	1.5

*Collagen for animals, bulk for plant
**Energy = Carbohydrates & Lipids

Supplementary Table S5: *Corrected isotopic food macronutrient values used in the Bayesian dietary modelling. *only used in the 8-food-group model, ** only used in the 5-food-group model.*

$\delta^{13}C$					
**Sources**		**Protein**	**uncertainty**	**Energy**	**uncertainty**
C_3 plants		-25.31	2	-22.81	2
C_4 plants		-12.45	1.5	-9.9	1.5
C_3 animals**		-22.74	1.5	-28.74	1.5
	Ovicaprids*	-23.01	2	-29.01	2
	Cattle*	-22.44	1.5	-28.44	1.5
	Pig*	-22.69	2	-28.69	2
	Poultry*	-21.15	2	-27.15	2
Marine		-11.9	4	-17.9	4
Freshwater		-20.29	7	-26.29	7
$\delta^{15}N$					
**Sources**		**Protein**		**Energy**	
C_3 plants		4.6	3.5	0	0
C_4 plants		6.85	4	0	0
C_3 animals**		4.95	2	0	0
	Ovicaprids*	4.1	2	0	0
	Cattle*	5.03	1.5	0	0
	Pig*	5.2	2.5	0	0
	Poultry*	10.51	2	0	0
Marine		11.98	3.5	0	0
Freshwater		11.51	4.5	0	0

Supplementary Table S6: *Bioavailable strontium sampling in South-Eastern France*[98]*; Source for Lithology and Representative Age: FR-BRGM via EGDI*[99] *different Lithology given by Willmes et al[98]

ID	Latitude	Longitude	$^{87}Sr/^{86}Sr$	SD	Lithology	Representative Age
France_T2.520	43.762551	7.213709	0.708252	0.000012	Sand	Pliocene
France_T2.521	43.752617	7.197852	0.708344	0.000026	Sand*	Holocene
France_T2.522	43.588757	6.915575	0.718970	0.000018	Orthogneiss	Ediacaran
France_T2.523	43.587844	6.899248	0.716033	0.000018	Paragneiss*	Cambrian
France_T2.525	43.511556	6.812746	0.715814	0.000014	Rhyolitoid	Permian
France_T2.526	43.487462	6.776065	0.722785	0.000019	Sandstone*	Permian
France_T2.527	43.354666	6.602892	0.719965	0.000014	Paragneiss*	Cambrian
France_T2.528	43.190991	6.374558	0.720855	0.000014	Orthogneiss	Ediacaran
France_T2.529	43.230114	5.806555	0.707578	0.000013	Impure Carbonate Sedimentary Rock*	Upper Cretaceous
France_T2.530	43.266368	5.738084	0.708912	0.000021	Limestone	Lower Cretaceous
France_T2.531	43.493350	6.49235	0.709084	0.000021	Orthogneiss*	Ediacaran
France_T2.532	43.538047	6.483706	0.709868	0.000018	Impure Carbonate Sedimentary Rock	Upper Triassic
France_T2.533	43.478794	6.042792	0.707896	0.000012	Limestone	Middle Jurassic
France_T2.534	43.509252	6.018364	0.708509	0.000016	Sandstone*	Triassic
France_T2.535	43.757998	6.079364	0.708250	0.000017	Sand*	Pliocene
France_T2.536	43.960824	6.186764	0.708490	0.000018	Impure Carbonate Sedimentary Rock*	Miocene
France_T2.537	44.110656	5.945934	0.707855	0.000011	Impure Carbonate Sedimentary Rock	Lower Cretaceous
France_T2.538	43.986893	5.902234	0.707698	0.000011	Impure Carbonate Sedimentary Rock*	Oligocene
France_T2.539	43.955541	5.864408	0.708525	0.000006	Impure Carbonate Sedimentary Rock	Miocene
France_T2.540	43.627488	5.48965	0.707873	0.000014	Impure Carbonate Sedimentary Rock	Oligocene

Supplementary Table S7: *Modelling Details and Parameters for AverageR*

**Employed Software**	**App/Model**
Data Search and Spatiotemporal Modelling (DSSM) (Version 24.10.1)	AverageR
**Uploaded File**	**Details**
Data employed for modelling	Strontium baseline dataset
Data selection	$^{87}Sr/^{86}Sr$ plant values for Liguria (this study) and selected 87Sr/86Sr from Willmes et al. 2014
**App/Model options**	**Selected Options**
Data Source	Uploaded Baseline data
File Type	.xlxs
Coordinate format	Decimal Degrees (e.g. "40.446" or "79.982")
Dependent Variable	87Sr/86Sr values
Dependent Variable Type	numeric
Uncertainty of dependent variable (optional)	blank
Longitude Variable	Longitude
Latitude Variable	Latitude
Site/ID (optional)	blank
Smooth Type	spherical
Number of Basic functions	20
Extrapolation Behaviour	Constant
Remove Model Outliers	No
Remove Data Outliers	No
Restrict Model Area	No
Bayesian Model	Yes
Varying Standard Error	No
Number of MCMC Iterations	1000
Number of Burnin Iterations	500
Number of MCMC chains	1
MCMC thinning (Keep every x-th Sample)	10
Amount of Smoothing	0.2
**Options for Results Visualisation**	**Selected Options**
Map Section Zoom/-x-Range in degrees Longitude	4
Map Centering	Default (0th Meridian)
Estimation Type	Mean
Show Model Estimates	Yes
Max Value of Range Dependent Variable	Default
Min Value of Range Dependent Variable	Default
Restrict Range Dependent Variable	No
Terrestrial	Yes
Show Locations on the Map	Yes

Supplementary Table S7 (continuation): *Modelling Details and Parameters for AverageR*

**Options for Results Visualisation**	**Selected Options**
Set colour and shape of location marks	Yes (fixed colour, black)
Show map grid	Yes
Show Map Scale	Yes
Show Plot Title	No
Show colour Scale Title	No
Show colour scale	Yes
Set axis labels	No
Set north arrow and scale size and position	No
Location Mark size	Default (1)
Apply Convex Hull	No
Mask/Show Output within Range of Points	Yes – 40km
Display up to Max Standard Error	Max (Default)
Colour palette	Red-Yellow-Green
Approximate number of colour levels	20
Plot Resolution	300
Scale point size by variable	No
Add location mark text labels	No

Supplementary Table S8: *Modelling Details and Parameters for LocateR*

**Employed Software**	**App/Model**
Data Search and Spatiotemporal Modelling (DSSM) (Version 24.10.1)	LocateR
**App/Model Options**	**Selected Options**
Data Source	Create Map
Selected Maps for Similarity Map	Baseline 87Sr/86Sr (previously created in and saved from AverageR)
Provide Data values	Individuals which fell outside of the local $^{87}Sr/^{86}Sr$ range (124, 445, 470, 823, 868)
Normalise values	Yes
Type of Normalisation	Max value equal to 1
Weight values	No
**Options for Results Visualisation**	**Selected Options**
Map Section Zoom/-x-Range in degrees Longitude	4
Map Centering	Default (0th Meridian)
Estimation Type	Mean
Show Model Estimates	Yes
Max Value of Range Dependent Variable	1
Min Value of Range Dependent Variable	0
Terrestrial	Yes
Show Map Grid	Yes
Show Map Scale	Yes
Show North Arrow	Yes
Show Plot Title	No
Show Color Scale Title	No
Show Color Scale	Yes
Set Axis labels	No
Set North arrow and scale size and position	No
Colour palette	Red-Yellow-Green
Show Data values in Plot	No
Reverse Colours	No
Smooth Colour Transition	No
Approximate Number of Colour Levels	10

Supplementary References

1. Lamboglia, N. Girolamo Rossi (1831-1914). *Rivista Ingauna e Intemelia* **19**, 1–4 (1964).
2. Barocelli, P. *Albintimilium*. (Accademia Nazionale dei Lincei, Roma, 1923).
3. Gandolfi, D. Luigi Bernabò Brea e Nino Lamboglia: due archeologi a confronto. *Rivista di Studi Liguri* **69**, 165–224 (2003).
4. Zanini, E. Rileggere Nino Lamboglia una generazione dopo. *Ligures* **6**, 182–190 (2008).
5. Martino, G. P., Chiocci, F., Bracco, C. & Occelli, F. *Albintimilium*, le necropoli. in *Archaeologia in Liguria 1, 2004-2005* 85–105 (De Ferrari, Genoa, 2008).
6. Pallarés, F. Tombe tardo-romane a Ventimiglia. *Rivista di Studi Liguri* **54**, 303–336 (1988).
7. Gambaro, L., Costa, S. & Chierici, S. Scavo di una sepoltura infantile nell'ambiente VII delle terme di *Albintimilium*. in *Archaeologia in Liguria 5: 2012-2013* (eds. Conventi, M., Del Lucchese, A. & Gardini, A.) 119–121 (SAGEP, Genoa, 2015).
8. Gandolfi, D. Il sepolcreto tardo antico della Porta Nord di Albintimilium. Aggiornamenti e prospettive di ricerca. in *Le Archeologie di Marilli. Miscellanea di studi in ricordo di Maria Maddalena Negro Ponzi Mancini* (ed. De Vingo, P.) 339–360 (Edizioni dell'Orso, Alessandria, 2018).
9. Arobba, D., Caramiello, R. & Martino, G. P. Indagi paleobotaniche su reperti di una tomba del IV-V secolo dC rinvenuta ad Albintimilium (Ventimiglia, Liguria). *Rivista di Studi Liguri* **63**, 323–336 (1999).
10. Vitale, L. Lo spazio degli infanti nei cimiteri tardo-antichi. Organizzazione e distribuzione spaziale fra ritualità e consuetudini sociali. in *Isole e terraferma nel*

primo cristianesimo. Identità locale ed interscambi culturali, religiosi e produttivi, Atti del XI Congresso Nazionale di Archeologia Cristiana, 23-27 settembre 2014, Cagliari (eds. Martorelli, R., Piras, A. & Spanu, P. G.) (PFS University Press, Cagliari, 2015).

11. Mennella, G. *Le Iscrizioni Romane Di Albintimilium*. (Istituto Internazionale di Studi Liguri, Bordighera, 2014).
12. Vertet, H. Les Techniques de fabrication des lampes en terre cuite du Centre de la Gaule, Recherches sur les ateliers de potiers de la Gaule centrale. *Revue Archéologique Sites* **3**, 20 (1983).
13. Buchi, E. *Lucerne Del Museo Di Aquileia 1: Lucerne Romane Con Marchio de Fabbrica*. (Associazione Nazionale per Aquileia, Aquileia, 1975).
14. Brando, M. La suppellettile da illuminazione. in *Diana Umbronensis a Scoglietto. Santuario, territorio e cultura materiale (200 a.C. - 550 d.C.)* (eds. Sebastiani, A., Chirico, E., Colombini, M. & Cygielman, M.) 114–224 (Archaeopress Archaeology, Oxford, 2015).
15. Deneauve, J. *Lampes de Carthage*. (Éditions du centre National de la Recherche Scientifique, Paris, 1969).
16. Béal, J. C. *Catalogue Des Objects de Tabletterie Du Musée de La Civilisation Gallo-Romaine de Lyon*. (Université Jean-Moulin, Lyon, 1983).
17. Chierici, S. Piccoli oggetti della quotidianità. Reperti in osso, metallo e altri materiali dagli scavi di *Albintimilium*. *Rivista di Studi Liguri* **80–81**, 175–216 (2014-2015).
18. Capitanio, M. Esame delle ossa umane della necropoli romana die Ventimiglia (II-IV sec. d. C.). Preprint at (1999).

19. Buikstra, J. E. & Ubelaker, D. Standards for data collection from human skeletal remains. *Arkansas archaeological survey research series* **44**, 18 (1994).

20. Klales, A. R., Ousley, S. D. & Vollner, J. M. A revised method of sexing the human innominate using Phenice's nonmetric traits and statistical methods. *American Journal of Physical Anthropology* **149**, 104–114 (2012).

21. Phenice, T. W. A newly developed visual method of sexing the os pubis. *American Journal of Physical Anthropology* **30**, 297–301 (1969).

22. Klales, A. R. MorphoPASSE: Morphological pelvis and skull sex estimation program. in *Sex Estimation of the Human Skeleton* 271–278 (Elsevier, 2020).

23. Walker, P. L. Sexing skulls using discriminant function analysis of visually assessed traits. *Am. J. Phys. Anthropol.* **136**, 39–50 (2008).

24. Krogman, W. M. & Isçan, M. Y. The human skeleton in forensic medicine. *C.C. Thomas, Springfield, IL* **15**, 202–208 (1986).

25. Moore, M. K. Chapter 4 - Sex Estimation and Assessment. in *Research Methods in Human Skeletal Biology* (eds. DiGangi, E. A. & Moore, M. K.) 91–116 (Academic Press, London, 2013).

26. Selliah, P. *et al.* Sex estimation of skeletons in middle and late adulthood: reliability of pelvic morphological traits and long bone metrics on an Italian skeletal collection. *Int. J. Legal Med.* **134**, 1683–1690 (2020).

27. Brooks, S. & Suchey, J. M. Skeletal age determination based on the os pubis: a comparison of the Acsádi-Nemeskéri and Suchey-Brooks methods. *Hum. Evol.* **5**, 227–238 (1990).

28. Nikita, E. *Osteoarchaeology: A Guide to the Macroscopic Study of Human Skeletal Remains.* (Academic Press, San Diego, CA, 2016).

29. Buckberry, J. L. & Chamberlain, A. T. Age estimation from the auricular surface of the ilium: A revised method. *Am. J. Phys. Anthropol.* **119**, 231–239 (2002).

30. Brothwell, D. R. *Digging Up Bones: The Excavation, Treatment, and Study of Human Skeletal Remains*. (Cornell University Press, Ithaca, NY, 1981).

31. Ousley, S. *et al. Transition Analysis 3 (TA3)*. (2021).

32. Milner, G. R. & Boldsen, J. L. Transition analysis: A validation study with known-age modern American skeletons. *Am. J. Phys. Anthropol.* **148**, 98–110 (2012).

33. AlQahtani, S. J., Hector, M. P. & Liversidge, H. M. Brief communication: The London atlas of human tooth development and eruption. *Am. J. Phys. Anthropol.* **142**, 481–490 (2010).

34. Liversidge, H. M., Dean, M. C. & Molleson, T. I. Increasing human tooth length between birth and 5.4 years. *Am. J. Phys. Anthropol.* **90**, 307–313 (1993).

35. Nikita, E., Karligkioti, A., Marklein, K. & Moutafi, I. Basic guidelines for the excavation and study of human skeletal remains. (2019).

36. Schaefer, M., Black, S. & Scheuer, L. *Juvenile Osteology*. (Academic Press, London, 2009).

37. Primeau, C., Friis, L., Sejrsen, B. & Lynnerup, N. A method for estimating age of medieval sub-adults from infancy to adulthood based on long bone length. *Am. J. Phys. Anthropol.* **159**, 135–145 (2016).

38. Scheuer, J. L., Musgrave, J. H. & Evans, S. P. The estimation of late fetal and perinatal age from limb bone length by linear and logarithmic regression. *Ann. Hum. Biol.* **7**, 257–265 (1980).

39. Fazekas, I. G. & Kósa, F. *Forensic Fetal Osteology*. (Akadémiai Kiadó, Budapest, 1978).

40. Scheuer, L. & Black, S. Development and ageing of the juvenile skeleton. *Human osteology in archaeology and forensic science* 9–22 (2000).

41. Laes, C. & Strubbe, J. *Youth in the Roman Empire: The Young and the Restless Years?* (Cambridge University Press, Cambridge, 2014).

42. Longin, R. New Method of Collagen Extraction for Radiocarbon Dating. *Nature* **230**, 241–242 (1971).

43. Ambrose, S. H. Preparation and characterization of bone and tooth collagen for isotopic analysis. *J. Archaeol. Sci.* **17**, 431–451 (1990).

44. DeNiro, M. J. Postmortem preservation and alteration of in vivo bone collagen isotope ratios in relation to palaeodietary reconstruction. *Nature* **317**, 806–809 (1985).

45. Guiry, E. J. & Szpak, P. Improved quality control criteria for stable carbon and nitrogen isotope measurements of ancient bone collagen. *Journal of Archaeological Science* **132**, 105416 (2021).

46. Coplen, T. B. Reporting of stable hydrogen, carbon, and oxygen isotopic abundances (Technical Report). *Pure Appl. Chem.* **66**, 273–276 (1994).

47. Chodová, D. & Tůmová, E. Insects in chicken nutrition. A review. *Agron. Res.* 440.2Kb (2020).

48. Coletta, L. D. *et al.* Barn vs. free-range chickens: Differences in their diets determined by stable isotopes. *Food Chem.* **131**, 155–160 (2012).

49. Grandal-d'Anglade, A., Gorobets, L., García-Vázquez, A. & Ivanoff, D. V. Feeding patterns and management of dogs and chickens from ancient to medieval sites of Ukraine: A stable isotope analysis. in *Archaeologies of Animal Movement. Animals on the Move* 59–72 (Springer International Publishing, Cham, 2021).

50. Ricci, A. Il porcile. in *Settefinestre: a villa schiavistica nell'Etruria romana. Vol. 2* (ed. Ricci, A.) 182–188 (Panini, Modena, 1985).
51. Pasquinucci, M. "Frequently the Winter Grazing Grounds are Many Miles Away from the Summer Ones" (Varro, de r.r. 2.2.9): A Review of Recent Historical, Anthropological and Archaeological Approaches to Transhumance in Central and Southern Italy. in *Transhumance: Papers from the International Association of Landscape Archaeology Conference, Newcastle upon Tyne, 2018* (eds. Bowden, M. & Herring, P.) 23–41 (Archaeopress, Oxford, 2021).
52. Trentacoste, A. *et al.* Isotopic Insights into Livestock Production in Roman Italy: Diet, Seasonality, and Mobility on an Imperial Estate. *Environ. Archaeol.* **0**, 1–23 (2023).
53. MacKinnon, M. High on the Hog: Linking Zooarchaeological, Literary, and Artistic Data for Pig Breeds in Roman Italy. *Am. J. Archaeol.* **105**, 649–673 (2001).
54. Trentacoste, A. *et al.* New trajectories or accelerating change? Zooarchaeological evidence for Roman transformation of animal husbandry in Northern Italy. *Archaeol. Anthropol. Sci.* **13**, 25 (2021).
55. Stroud, E., Charles, M., Bogaard, A. & Hamerow, H. Turning up the heat: Assessing the impact of charring regime on the morphology and stable isotopic values of cereal grains. *J. Archaeol. Sci.* **153**, 105754 (2023).
56. Fernandes, R., Nadeau, M.-J. & Grootes, P. M. Macronutrient-based model for dietary carbon routing in bone collagen and bioapatite. *Archaeol Anthropol Sci* **4**, 291–301 (2012).
57. Sołtysiak, A. & Fernandes, R. Much ado about nothing: assessing the impact of the 4.2 kya event on human subsistence patterns in northern Mesopotamia using stable isotope analysis. *Antiquity* **95**, 1145–1160 (2021).

58. Fernandes, R., Millard, A. R., Brabec, M., Nadeau, M.-J. & Grootes, P. Food reconstruction using isotopic transferred signals (FRUITS): a Bayesian model for diet reconstruction. *PLoS One* **9**, e87436 (2014).
59. Fernandes, R., Grootes, P., Nadeau, M. & Nehlich, O. Quantitative diet reconstruction of a Neolithic population using a Bayesian mixing model (FRUITS): The case study of Ostorf (Germany). *Am. J. Phys. Anthropol.* **158**, 325–340 (2015).
60. Bownes, J. M., Ascough, P. L., Cook, G. T., Murray, I. & Bonsall, C. Using stable isotopes and a Bayesian mixing model (FRUITS) to investigate diet at the early Neolithic site of Carding Mill Bay, Scotland. *Radiocarbon* **59**, 1275–1294 (2017).
61. Varano, S. *et al.* The edge of the Empire: diet characterization of medieval Rome through stable isotope analysis. *Archaeol. Anthropol. Sci.* **12**, 196 (2020).
62. Cocozza, C., Cirelli, E., Groß, M., Teegen, W. R. & Fernandes, R. Compendium Isotoporum Medii Aevi (CIMA). https://doi.org/10.48493/s9nf-1q80 (2021).
63. Cocozza, C., Cirelli, E., Groß, M., Teegen, W.-R. & Fernandes, R. Presenting the Compendium Isotoporum Medii Aevi, a Multi-Isotope Database for Medieval Europe. *Sci Data* **9**, 354 (2022).
64. Formichella, G., Soncin, S. & Cocozza, C. Isotòpia: A Stable Isotope Database for Classical Antiquity. https://doi.org/10.48493/m0m0-b436 (2023).
65. Formichella, G. *et al.* Introducing Isotòpia: A stable isotope database for Classical Antiquity. *PLoS One* **19**, e0293717 (2024).
66. Alagich, R., Gardeisen, A., Alonso, N., Rovira, N. & Bogaard, A. Using stable isotopes and functional weed ecology to explore social differences in early urban contexts: The case of Lattara in mediterranean France. *J. Archaeol. Sci.* **93**, 135–149 (2018).

67. Gismondi, A. *et al.* Back to the roots: dental calculus analysis of the first documented case of coeliac disease. *Archaeol. Anthropol. Sci.* **12**, 6 (2020).
68. O'Connell, T. C. *et al.* Living and dying at the Portus Romae. *Antiquity* **93**, 719–734 (2019).
69. Arobba, D. Macroresti botanici rinvenuti nei livelli tardoantichi e medievali del battistero della cattedrale di Ventimiglia. *Rivista di Studi Liguri* **66**, 197–212 (2001).
70. Arobba, D., Caramiello, R. & Martino, G. P. Il contributo delle analisi archeobotaniche per la storia del paesaggio agrario della città romana di Albintimilium (Ventimiglia). *Rivista di Studi Liguri* **70**, 283–206 (2005).
71. Arobba, D., Bulgarelli, F., Siniscalco, C. & Caramiello, R. Roman landscape and agriculture on the Ligurian coast through macro and microremains from a Vada Sabatia well (Vado Ligure, Italy). *Environ. Archaeol.* **18**, 114–131 (2013).
72. Nitsch, E. *et al.* A bottom-up view of food surplus: using stable carbon and nitrogen isotope analysis to investigate agricultural strategies and diet at Bronze Age Archontiko and Thessaloniki Toumba, northern Greece. *World Archaeol.* **49**, 105–137 (2017).
73. Cocozza, C. *et al.* A Bayesian multi-proxy contribution to the socioeconomic, political, and cultural history of late medieval Capitanata (southern Italy). *Scientific Reports* **13**, 4078 (2023).
74. Mion, L. *et al.* Contribution to Mediterranean medieval dietary studies: Stable carbon and nitrogen isotope data of marine and catadromous fish from Provence (9th–14th CE). *Data in Brief* **41**, 108016 (2022).

75. Grasso, L. Anfore. in *Dalla villa al villaggio. Corti: scavo di un sito archeologico di età romana e altomedievale lungo il metanodotto del Ponente ligure* (ed. Massabò, B.) 155–170 (Erga Edizioni, Genoa, 1999).
76. Carabia, A. Space, population, and economy in a frontier region: Liguria in the context of the western Byzantine provinces (500-700 CE). (University of Birmingham, 2022).
77. Gandolfi, D. Ceramiche fini di importazione di VI-VII secolo in Liguria. L'esempio di Ventimiglia, Albenga e Luni. in *Ceramica in Italia: VI-VII secolo* (ed. Saguì, L.) 253–274 (All'Insegna del Giglio, Florence, 1998).
78. De Vingo, P. Liguria in Late Antiquity and the Early Middle Ages: its trade relations with the Western and Eastern Mediterranean Sea. in *LRCW I. Late Roman Coarse Wares Cooking Wares and Amphorae in the Mediterranean* (eds. Gurt i Esparraguera, J. M., Buxeda i Garrigós, J. & Cau Ontiveros, M. A.) 341–354 (BAR, Oxford, 2005).
79. Tinterri, D. Trading amphorae in late roman Liguria (AD 400-700). in *LRCW 4.The Mediterranean: a Market Without Frontiers* (eds. Poulou-Papadimitriou, N., Nodarou, E. & Kilikoglou, V.) 957–983 (Archaeopress, Oxford, 2014).
80. Gandolfi, D., Martino, G. P. & Occelli, F. Vintimilio plagia. Approdi viabilità e merci nel territorio del municipio di *Albintimilium*. in *Porti antichi, retroterra produttivi, strutture, rotte, merci* (eds. Pasquinucci, M. & Facella, A.) 353–356 (Pisa University Press, Pisa, 2023).
81. Soncin, S. *et al.* High-resolution dietary reconstruction of victims of the 79 CE Vesuvius eruption at Herculaneum by compound-specific isotope analysis. *Sci. Adv.* **7**, eabg5791 (2021).

82. De Ruiter, H., Kastner, T. & Nonhebel, S. European dietary patterns and their associated land use: Variation between and within countries. *Food Policy* **44**, 158–166 (2014).

83. Bertani, A. & Marenco, A. *Come Vivevamo: Casa, Cibo e Igiene Dei Contadini in Liguria a Fine Ottocento: Dagli Atti Dell'inchiesta Agraria (1877-1886) Diretta Da Stefano Jacini*. (Pentàgora Edizione, Savona, 2021).

84. Arobba, D., Caramiello, R. & Firpo, M. Contributi paleobotanici alla storia dell'evoluzione di una pianura costiera: il caso di Albenga. in *I Liguri. Un Antico Popolo Europeo tra Alpi e Mediterraneo.* (eds. De Marinis, R. C. & Spadea, G.) 76–78 (Skira, Ginevra, Milano, 2004).

85. Ruas, M.-P. Cultures et moissons à Fiorentino : étude des semences carbonisées. in *Fiorentino ville désertée nel contesto della Capitanata medievale (Ricerche 1982-1993)* (eds. Calò Mariani, M. S., Piponniier, F., Beck, P. & Laganara, C.) 541–565 (École Française de Rome, Rome, 2012).

86. Favia, P. *et al.* Modelli di trattamento degli alimenti in un contesto castrense medievale: la cucina e la dispensa della rocca di Montecorvino. *Facta. A Journal of late Roman, Medieval and Post Medieval Material Cultural Studies* **8**, 25–55 (2014).

87. Di Francescantonio, L. I reperti faunistici. in *Il teatro romano di Albintimilium: restauri e ricerche (2011-2017)* (ed. Gambaro, L.) 54–56 (SAGEP, Genoa, 2021).

88. Congès, G., Leguilloux, M. & Brien-Poitevin, F. Un dépotoir de l'Antiquité tardive dans le quartier de l'Esplanade à Arles. *Revue archéologique de Narbonnaise* **24**, 201–234 (1991).

89. Di Francescantonio, L. Reperti Faunistici. in *Dalla villa al villaggio. Corti: scavo di un sito archeologico di età romana e altomedievale lungo il metanodotto del Ponente ligure* (ed. Massabò, B.) 198–202 (Erga Edizioni, Genoa, 1999).
90. Marazzo, D. & Spinetti, A. Lo studio archeozoologico. in *San Paragorio di Noli. Le fasi del complesso di culto e l'insediamento circostante dalle origini all'XI secolo* (ed. Frondoni, A.) 311–326 (All'Insegna del Giglio, Florence, 2018).
91. Corbino, C. A., Minniti, C., J., D. E. G. M. & Albarella, U. The role of chicken in the medieval food system: evidence from Central Italy. *TMA* **56**, 50–57 (2017).
92. Guarini, E. *Albintimilium*: gli animali, il cibo e l'industria su osso. *Archeologia in Liguria* **I**, 376–377 (2008).
93. Bisio, E. I reperti faunistici. in *Filattiera-Sorano: gli insediamenti sul dosso della Pieve e altre ricerche* (ed. Giannichedda, E.) 191–195 (All'Insegna del Giglio, Florence, 2010).
94. Giovinazzo, R. I reperti faunistici. in *Filattiera-Sorano: L'insediamento di età romana e tardoantica. Scavi 1986-1995* (ed. Giannichedda, E.) 196–197 (All'Insegna del Giglio, Florence, 1998).
95. Massy, K., Friedrich, R., Mittnik, A. & Stockhammer, P. W. Pedigree-based Bayesian modelling of radiocarbon dates. *PLoS One* **17**, e0270374 (2022).
96. Gnecchi-Ruscone, G. A. *et al.* Network of large pedigrees reveals social practices of Avar communities. *Nature* 1–8 (2024).
97. Ramsey, C. B. Radiocarbon calibration and analysis of stratigraphy: the OxCal program. *Radiocarbon* **37**, 425–430 (1995).
98. Willmes, M. *et al.* The IRHUM (Isotopic Reconstruction of Human Migration) database – bioavailable strontium isotope ratios for geochemical fingerprinting in France. *Earth Syst. Sci. Data* **6**, 117–122 (2014).

99. EGDI 1:1 Million pan-european Surface Geology. (2022).
